# Recyclable anhydride catalyst for H_2_O_2_ oxidation: *N*-oxidation of pyridine derivatives†

**DOI:** 10.1039/d0ra00265h

**Published:** 2020-03-03

**Authors:** Ghellyn Gajeles, Se Mi Kim, Jong-Cheol Yoo, Kyung-Koo Lee, Sang Hee Lee

**Affiliations:** Department of Chemistry, Kunsan National University Daehakro 558 Gunsan Jeonbuk 54150 South Korea leesh@kunsan.ac.kr; Lotte Chemical Research Institute, S&E Management 115 Gajeongbuk-ro Daejeon 34110 South Korea

## Abstract

The catalytic efficiency and recyclability of poly(maleic anhydride-*alt*-1-octadecene) (Od-MA) and poly(maleic anhydride-*alt*-1-isobutylene) (Bu-MA) were evaluated for use in the development of a metal-free, reusable catalyst for the oxidation of pyridines to pyridine *N*-oxides in the presence of H_2_O_2_. The Od-MA catalyst was easily recovered *via* filtration with recovery yields exceeding 99.8%. The catalyst retained its activity after multiple uses and did not require any treatment for reuse. The Od-MA and H_2_O_2_ catalytic system described herein is eco-friendly, operationally simple, and cost-effective; thus, it is industrially applicable. Od-MA and H_2_O_2_ could potentially be used in place of percarboxylic acid as an oxidant in a wide range of oxidation reactions.

## Abstract 2

Od-MA polymer is developed as the first metal-free, recyclable anhydride catalyst used for the *N*-oxidation of pyridines with H_2_O_2._

## Graphical abstract

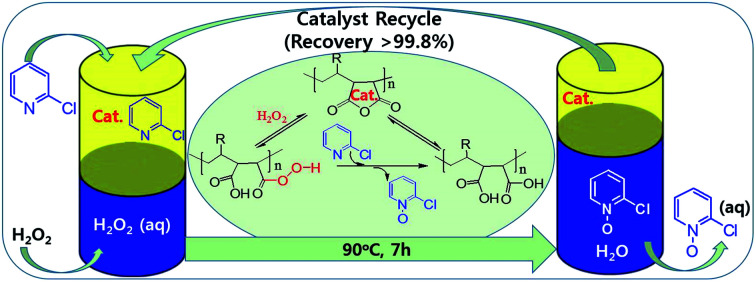

## 1 Introduction

Various metal-catalyzed, hydrogen peroxide-based oxidation methods have been developed for the synthesis of a variety of chemicals.^1–3^ Peroxides incorporating group VI elements, such as vanadium, tungsten, and molybdenum,^4–8^ are potent inorganic catalysts. Titanosilicate is an efficient and recyclable catalyst for the oxidation of a variety of organic compounds in the presence of H_2_O_2_.^9^ Unfortunately, inorganic catalysts must be removed from reaction mixtures, resulting in environmental problems due to the risk of leaching.

Recently, a number of metal-free H_2_O_2_ oxidation protocols have been developed. Ammonium salt,^10^ ionic liquid,^11^ DABCO tribromide,^12^ and porous carbon^13^ were used for activation of H_2_O_2_. To improve the oxidation ability of H_2_O_2_ in the absence of a metal catalyst, carboxylic acids or anhydrides have also been used as mediators because the corresponding percarboxylic acids are more reactive than H_2_O_2_. Many percarboxylic acids are prepared *in situ* or immediately before use because they are unstable. Occasionally, peracids are formed from H_2_O_2_ and the corresponding carboxylic acid.^14,15^ However, weak carboxylic acids, such as acetic acid or benzoic acid, require a strong acid catalyst for conversion to percarboxylic acid.^16,17^ As alternatives to carboxylic acids, carboxylic anhydrides have been used for the *in situ* production of percarboxylic acid with H_2_O_2_.^18,19^ Acetic anhydride,^20^ trifluoroacetic anhydride,^21,22^ maleic anhydride,^23,24^ and phthalic anhydride^25^ have been used as mediators in oxidation reactions based on H_2_O_2_. Taddei *et al.*^26^ employed polymer-supported phthalic anhydride for alkene epoxidation but has never mentioned about the reusability of the polymer catalyst.

Pyridine *N*-oxides are useful synthetic intermediates, protecting groups, auxiliary agents, oxidants, and ligands in metal complexes and catalysts.^27–34^ 2-Chloropyridine-*N*-oxide (CPNO) is of particular interest because it is the precursor to zinc-2-pyridinethiol-1-oxide, an antibacterial and antifungal agent used in many over-the-counter creams, lotions, soaps, and shampoos.^35^ Anhydride-H_2_O_2_ oxidizes pyridine derivatives to their corresponding *N*-oxides.^21^ However, the recyclability of anhydride-H_2_O_2_ catalysts is unknown. A recyclable anhydride mediator is highly desirable for industrial processes that rely on *N*-oxidation using H_2_O_2_. In an effort to develop an industrially applicable process, this study evaluated polymer-supported maleic anhydride as a metal-free, reusable mediator for the *N*-oxidation of pyridine derivatives with H_2_O_2_.

## 2 Experimental

### 2.1 Materials and instruments

Pyridine derivatives and H_2_O_2_ (H0300, 35%) were purchased from Tokyo Chemical Industry and Samchun Chemicals, respectively. Poly(isobutylene-*alt*-maleic anhydride) (531278), and poly(maleic anhydride-*alt*-1-octadecene) (419117), were purchased from Sigma Aldrich. ^1^H NMR and ^13^C NMR spectral measurements were obtained from a 500 MHz Agilent (Varian) VNMRS spectrometer with tetramethylsilane as the reference. FTIR spectra of the catalysts were recorded on a Jasco FT/IR-6300 Spectrometer. Selectivity and conversion yield of CPNO were determined using a Gas Chromatography/Mass Spectrometer (GC/MS, Varian-320MS).

### 2.2 Catalytic reactions

Oxidation of pyridine was carried out under vigorous stirring in a 50 ml glass flask connected to a cooling condenser. In a typical run, 1.14 g (10 mmol) of 2-chloropyridine (2CP), 2 ml of H_2_O_2_ (34 wt% aqueous solution), 2 ml H_2_O, and 0.76 g (2 mmol) of Od-MA were mixed in the flask and heated at different temperatures under agitation for 7 h. After removal of the catalyst by filtration, the aqueous mixture containing CPNO and recovered 2CP was subjected to GC analysis to determine the CPNO conversion yield. For the analysis of NMR spectrum of CPNO, the remaining H_2_O_2_ in aqueous mixture was quenched with Na_2_S_2_O_3_ and washed with hexane to remove unreacted pyridine. After evaporation of water, the residue was dissolved in CH_2_Cl_2_ and filtered out insoluble materials. The filtrate was concentrated to obtain CPNO. *δ*^1^H NMR (500 MHz, CdCl_3_): 7.28–7.32 (2H, m, Ar–H), 7.55–7.58 (1H, m, Ar–H), 8.40–8.41 (1H, m, Ar–H) ppm. *δ*^13^C NMR (125 MHz, CdCl_3_ w/DMSO): 123.8, 126.0, 126.9, 140.3, 141.5 ppm.

### 2.3 Measurement of opening rate of anhydride by H_2_O_2_

During vigorous stirring of the reaction mixture of CP (10 mmol), Od-MA (2 mmol) and H_2_O_2_ (20 mmol, 15%) at room temperature, 0.5 ml of the reaction mixture was taken at each time (after 1 h, 2 h, and 3 h), diluted with 10 ml of CH_3_CN and filtered to obtain solid Od-MA. After washing with water followed by CH_3_CN, the recovered Od-MA was dried under vacuum (0.1 torr) at room temperature for 3 h and used as an IR sample. In the IR spectrum of the recovered Od-MA, the decrease of anhydride signals at 1859 and 1780 cm^−1^ were monitored.

### 2.4 Determination of recovery yield of catalyst

After oxidation reaction, used Od-MA was filtered off and the aqueous filtrate was concentrated using rotary evaporator to obtain the mixture of pyridine *N*-oxide (PNO), recovered 2-chloropyridine and water-extracted Od-MA catalyst. In ^1^H NMR (DMSO-*d*_6_) of the mixture, the mole ratio of Od-MA/CPNO was measured to determine the amount of extracted Od-MA in aqueous mixture.

### 2.5 Conversion of Od-MA to Od-MA-CO_2_Na, hydrolyzed-Od-MA, and Od-MA-CO_2_Me

#### Od-MA-CO_2_Na

A solution of 1.14 g (10 mmol) of Od-MA and 1.2 g (30 mmol) of NaOH in deionized water (30 ml) was stirred overnight at room temperature and then refluxed for 4 h. The reaction mixture was concentrated to get Od-MA-CO_2_Na.

#### Od-MA-hydrolyzed

1.0 g of Od-MA-CO_2_Na was dissolved in deionized water (20 ml) and acidified with HCl. After filtration, the solid was washed with water and dried for 6 h under vacuum at 70 °C to obtain the hydrolyzed-Od-MA as a white solid.

#### Od-MA-CO_2_Me

A solution of 0.5 g of hydrolyzed-Od-MA in MeOH–toluene (30 ml, 1 : 1) was refluxed for 6 h. The reaction mixture was concentrated to obtain Od-MA-CO_2_Me as a white solid.

### 2.6 Synthesis of zinc pyrithione from 2CP

A solution of 11.3 g (0.1 mol) of 2CP, 34 ml (1.5 eq.) of H_2_O_2_ (15%) and 7.6 g (0.2 eq.) of Od-MA were stirred using mechanical stirrer for 5 h at 80 °C. After cooling to room temperature, the aqueous layer was decanted and recovered Od-MA was washed with water (10 ml × 2). Aqueous layers are combined and unreacted 2CP was extracted with toluene (10 ml × 2). After addition of 15 ml of Na_2_S_2_O_3_ (3 M), aqueous layer was stirred for 30 min at room temperature to quench the remaining H_2_O_2_. 6.12 g (0.11 mol) of NaSH (0.11 mol) and 4.37 g of NaOH were added and stirred for 3 h at 80 °C. After cooling the reaction mixture, concentrated HCl was added to adjust pH to 6.5. Resulted floating material was removed by filtration and nitrogen gas was bubbled for 30 min through the reaction mixture to remove H_2_S gas (from remaining NaSH + HCl). 20 ml of ZnSO_4_ solution (2 M) was added dropwise to precipitate zinc pyrithione. After filtration, the solid product was dried under vacuum at 80 °C to obtain 12.5 g of zinc pyrithione as a white solid. (94% from CPNO). *δ*^1^H NMR (500 MHz, DMSO): 6.99–7.02 (1H, td, *J* = 2 Hz, 7 Hz, Ar–H), 7.23–7.26 (1H, m, Ar–H), 7.60–7.62 (1H × 2, dd, *J* = 1.5, 8.5 Hz, Ar–H), 8.42–8.44 (1H × 2, dd, *J* = 1.0, 6.5 Hz, Ar–H) ppm. *δ*^13^C NMR (125 MHz, DMSO): 117.9, 128.4, 129.3, 137.3, 159.3 ppm.

## 3 Results and discussion

### 3.1 Polymer catalysts

**Fig. 1:**
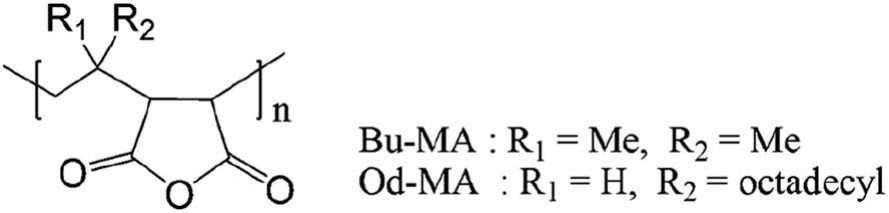
Molecular structures of the catalysts.

Two commercially available polymer catalysts were evaluated for the oxidation of 2-chloropyridine (CP) to its *N*-oxide (CPNO) in the presence of H_2_O_2_. Both polymers, poly (maleic anhydride-*alt*-1-isobutylene) (Bu-MA) or poly (maleic anhydride-*alt*-1-octadecene) (Od-MA) (Fig. 1), are alternating copolymers that contain repeating units of maleic anhydride and vinyl monomers. The average *M*_w_ of Bu-MA and Od-MA were 6000 and 40 000, respectively.

### 3.2 Catalytic reactions

**Scheme 1:**
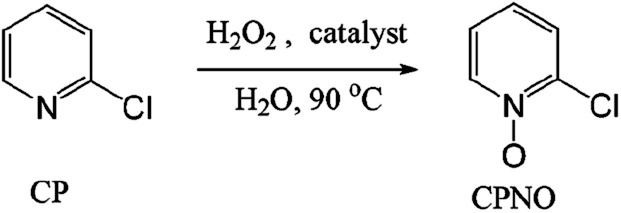
Catalytic oxidation of CP to CPNO using H_2_O_2_ and an anhydride catalyst.

The catalytic oxidation of CP to CPNO using 0.2 eq. of Od-MA or Bu-MA as a catalyst was carried out in 15% aqueous H_2_O_2_ over the course of 7 h at 90 °C (Scheme 1). At the beginning of the reaction, both catalysts were soluble in CP and the reaction mixture was a two-phase system. However, the catalyst precipitated as the reaction proceeded and the amount of CP decreased, resulting in a clear aqueous solution. After the reaction, the polymer catalyst was recovered *via* filtration and washed with CH_3_CN. The CH_3_CN extract and aqueous filtrate were combined; CPNO conversion yields were then determined by GC. The results are summarised in Table 1. As presented, a conventional system using maleic anhydride (MA) as a catalyst gave a lower yield of *N*-oxide compared to Od-MA. The selectivity of CPNO was >98% regardless of the reaction time, temperature, or amount of catalyst used.

**Table 1:** Catalytic oxidation of CP to CPNO using H_2_O_2_

Catalyst^a^	Yield^b^	Selectivity^b^
Od-MA	93	>98
Bu-MA	79	>98
MA	86	>98

a Reaction conditions: catalyst (0.2 eq.), H_2_O_2_ (2.0 eq.), 90 °C, 7 h.

b Determined by GC.

### 3.3 Mechanistic investigation of the catalytic activity of Od-MA

**Fig. 2:**
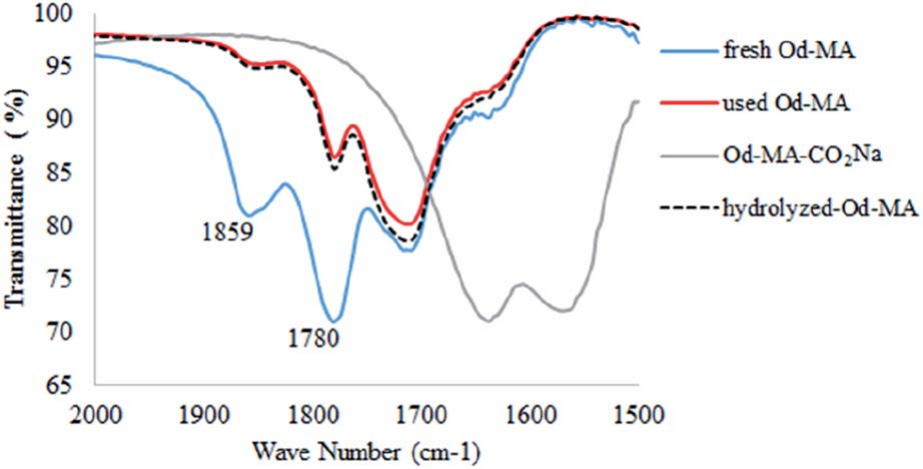
IR spectrum of used Od-MA and hydrolyzed Od-MA.

In the FTIR spectrum of recovered Od-MA, double *ν*C=O stretching bands at 1859 cm^−1^ and 1780 cm^−1^ were assigned to residual anhydride (Fig. 2).^36^ Repeated use of Od-MA did not change the amount of residual anhydride, presumably because of an equilibrium between the dicarboxylic acid and anhydride forms of Od-MA.

**Scheme 2:**
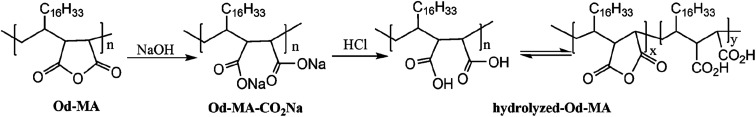
Hydrolysis and equilibrium between carboxylic acid and anhydride forms of Od-MA.

To examine the equilibrium between its dicarboxylic acid and anhydride forms, fresh Od-MA was hydrolyzed to Od-MA-CO_2_Na using NaOH and then acidified with HCl to obtain hydrolyzed-Od-MA (Scheme 2). Complete hydrolysis of Od-MA was confirmed *via* IR spectroscopy of Od-MA-CO_2_Na (Fig. 2). Absorption bands at 1859 cm^−1^ and 1780 cm^−1^ disappeared in the FTIR spectrum of Od-MA-CO_2_Na, indicating complete hydrolysis of the anhydride groups. However, when Od-MA-CO_2_Na was acidified, the IR spectrum of the resulting hydrolyzed-Od-MA was nearly identical to that of used Od-MA. Characteristic anhydride bands reappeared in the IR spectrum of hydrolyzed-Od-MA, indicating a regeneration of anhydride from carboxylic acid. These results demonstrated an equilibrium between the carboxylic acid and anhydride forms of hydrolyzed-Od-MA. The percentage of anhydride present was roughly estimated as 29–34% of the repeating units *via* IR spectral analysis (ESI, S1†).

**Fig. 3:**
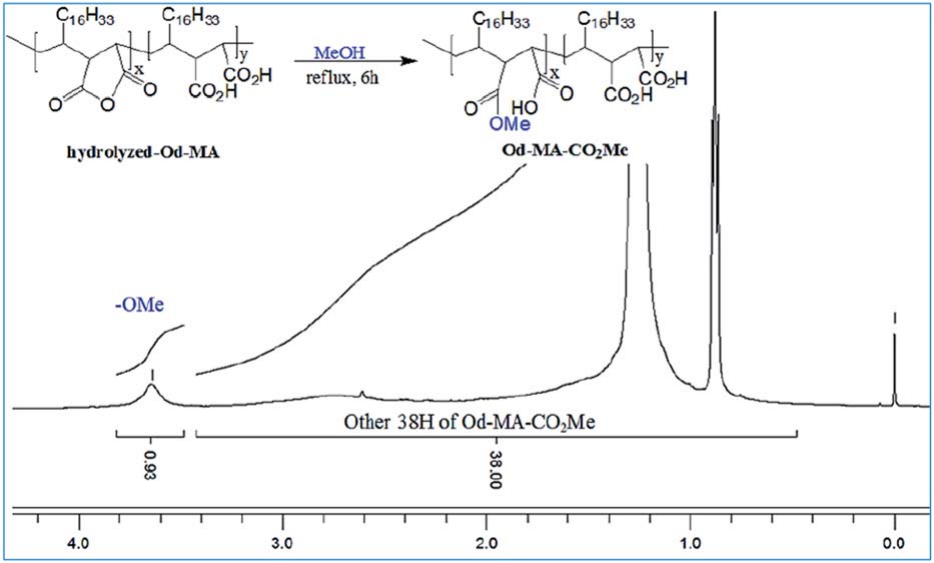
^1^H NMR of Od-MA-CO_2_Me obtained from hydrolyzed Od-MA.

To determine the amount of anhydride present in hydrolyzed-Od-MA, it was converted to Od-MA-CO_2_Me *via* methanolysis and then subjected to ^1^H NMR analysis. Since carboxylic acid is inert to methanolysis without acid catalyst, only the anhydride is converted to methyl ester. Thus, we presumed that the amount of Od-MA-CO_2_Me methyl ester represented the remaining anhydride in hydrolyzed-Od-MA. In the ^1^H NMR spectrum of Od-MA-CO_2_Me, we estimated the amount of anhydride to be 32% of the repeating units, based on the peak area ratio between the methyl ester protons (3.64 ppm) and other protons (38H, 0.5–3.4 ppm) of Od-MA (Fig. 3).

**Fig. 4:**
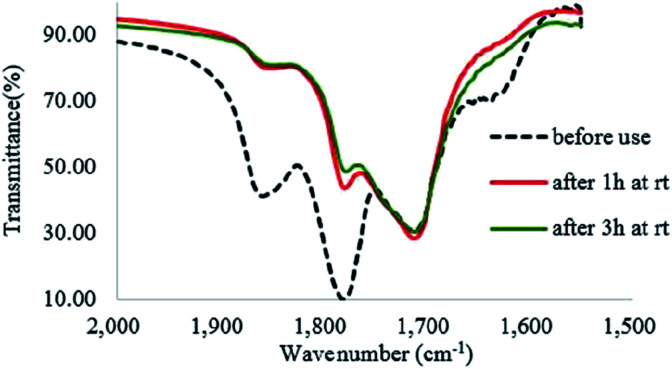
IR spectrum of recovered Od-MA during the oxidation of CP.

**Scheme 3:**
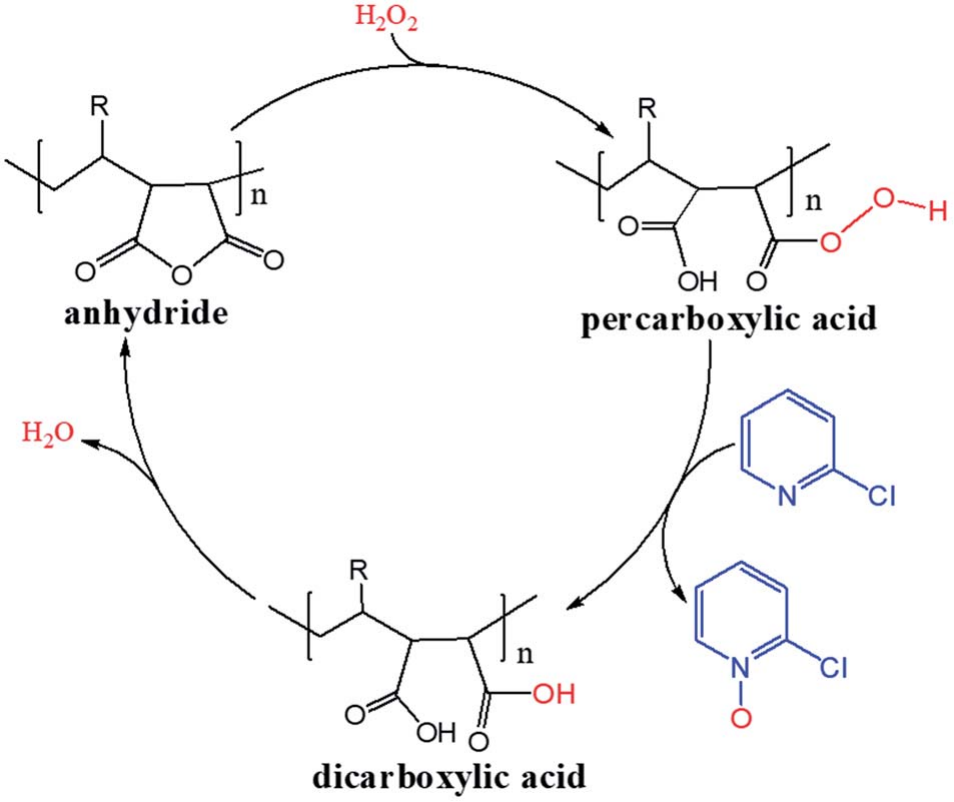
Proposed mechanism for Od-MA-catalysed oxidation of pyridines in the presence of H_2_O_2_.

H_2_O_2_ is a much stronger nucleophile than water^37^ and is highly reactive toward anhydrides.^38^ The anhydride of Od-MA is rapidly converted to percarboxylic acid upon reaction with H_2_O_2_. However, it is difficult to determine the stretching bands of percarboxylic acid in the IR spectrum of the used Od-MA probably because CO–OOH can be rapidly converted to anhydride due to unstable –OOH which is a very good leaving group. Hence, the formation of percarboxylic acid was determined by monitoring the decreasing intensity of anhydride bands in the FTIR spectrum (1780 cm^−1^ and 1859 cm^−1^) of recovered Od-MA in the oxidation reaction mixture. The conversion of anhydride to percarboxylic acid was sufficiently rapid that more than 70% of the Od-MA anhydride was opened by H_2_O_2_ within 1 h at room temperature, as shown by the decreased in the intensity of anhydride signals in Fig. 4. However, the yield of CPNO was only 1.1%. These data indicate that the rate-limiting step in the Od-MA catalysis reaction is the oxidation of CP by percarboxylic acid. Based on the regeneration of anhydride and its rapid conversion to percarboxylic acid, we propose the catalytic mechanism for Od-MA in Scheme 3. As illustrated, H_2_O_2_ converts the anhydride unit of Od-MA to percarboxylic acid, which plays as an active catalyst for the oxidation of pyridines to pyridine *N*-oxides. During the reaction, percarboxylic acid is converted to dicarboxylic acid, and the latter is also oxidized back to percarboxylic acid by H_2_O_2_.

**Scheme 4:**
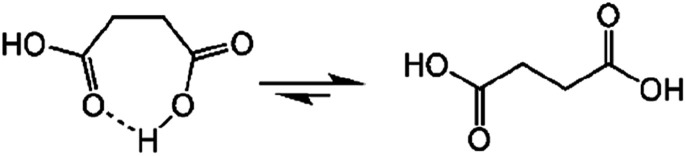
Conformational flexibility of succinic acid in water.

**Scheme 5:**
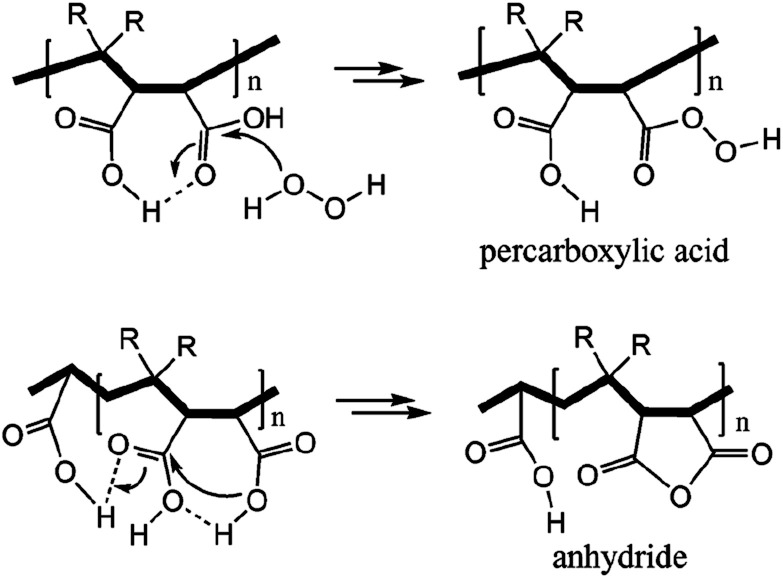
Formation of anhydride and percarboxylic acid facilitated by intramolecular hydrogen bonding.

Succinic anhydride is a repeating unit in Od-MA. When succinic anhydride was used as a catalyst, instead of the Od-MA polymer, the oxidation of CP provided a CPNO yield of only 6%. The same reaction conditions using Od-MA yielded 93%. This marked difference is likely due to the conformational rigidity of the Od-MA polymer, relative to the conformational flexibility of succinic acid. In aqueous media, intramolecular hydrogen bonding of succinic acid is unfavorable because of hydrogen bonding between succinic acid and water (Scheme 4).^39^ Conversely, restricted movement of the polymer backbone and the hydrophobic microenvironment of Od-MA favors hydrogen bond formation,^40^ which in turn facilitates nucleophilic attack by H_2_O_2_ to form percarboxylic acid or anhydride formation (Scheme 5). The hydrophobicity of Od-MA would also facilitate the approach of CP to catalytic sites.

### 3.4 Effects of reaction parameters on reaction rate

**Fig. 5:**
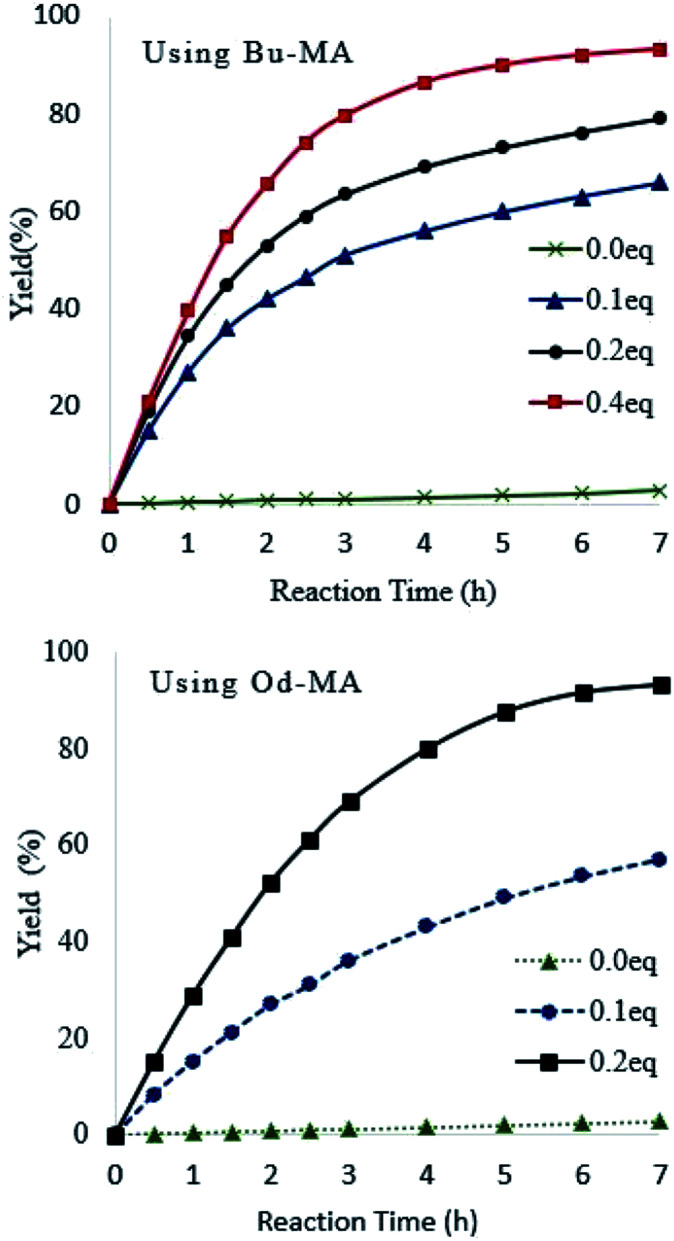
Effects of catalyst amount on reaction rates and yield. Reaction conditions: CP (10 mmol), aqueous H_2_O_2_ (20 mmol, 15%), 90 °C.

Catalyst equivalents were calculated based on the molecular weights of repeating units in the polymer catalysts (Od-MA = 350.54 g mol^−1^; Bu-MA = 154.17 g mol^−1^). As shown in Fig. 5, reaction rates increased concomitantly with the amount of catalyst present. With 0.2 eq. of Od-MA, the yield of CPNO reached 93% within 7 h at 90 °C. In contrast, the same amount of Bu-MA resulted in a 79% yield of CPNO. Without catalyst, the yield of CPNO was only 2.7% under the same conditions.

**Fig. 6:**
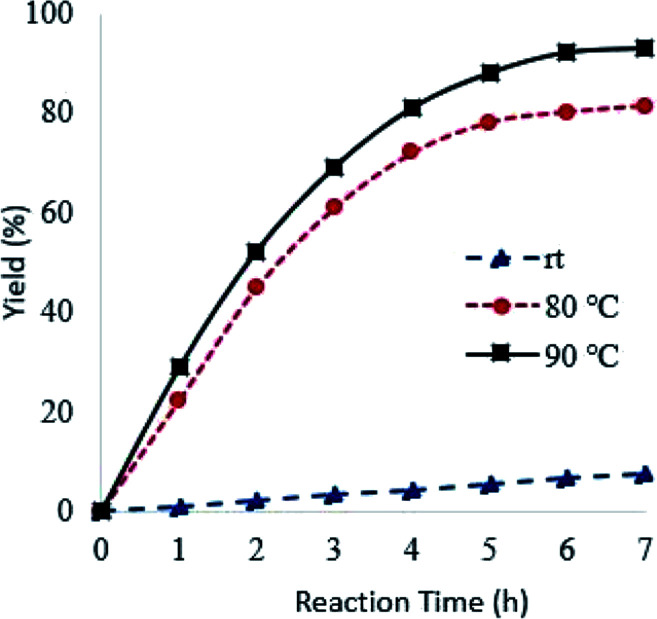
Effects of reaction temperature on reaction rates. Reaction conditions: CP (10 mmol), aqueous H_2_O_2_ (20 mmol, 15%), 0.2 eq. of Od-MA.

Reaction temperature had a marked effect on the oxidation of pyridine using Od-MA. As shown in Fig. 6, the conversion of pyridine increased concomitantly with increasing temperature, reaching >90% at 90 °C over 7 h. In contrast, the oxidation reaction proceeded slowly at room temperature and produced a CPNO yield of only 7.6% over the same time period.

Oxidation rates also depend on the electron density of pyridine derivatives. The effects of substituents on the *N*-oxidation rates of selected pyridine derivatives are summarised in Table 2. Electron withdrawing substituents reduce the nucleophilicity of pyridine derivatives undergoing *N*-oxidation.^41,42^ Therefore, a Preyssler catalyst is ineffective for the *N*-oxidation of 2-bromopyridine. Picolines, which are slightly more basic, were less reactive than pyridine because *N*-protonation was an inhibiting factor in *N*-oxidation using a TS-1 catalyst.^43^ We, therefore, expected very low reactivity with picolines because Od-MA contains a carboxylic acid moiety that can efficiently protonate picolines. The relative oxidation reactivity of pyridine derivatives was examined at 70 °C. After 5 h, conversion yields were 31–54%; all substrates showed similar reactivities. *N*-oxidations were nearly completed within 7 h at 90 °C.

**Table 2:** *N*-oxidation of pyridine derivatives using Od MA^a^

Entry	Substrate	Yield^b^ (%)	
		70 °C, 5 h	90 °C, 7 h
1	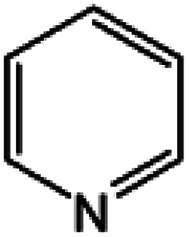	54	98
2	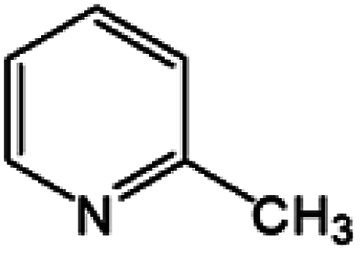	40	98
3	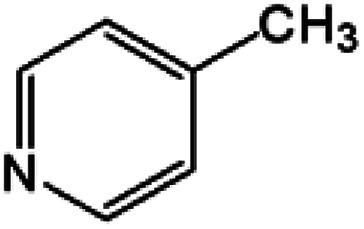	39	99
4	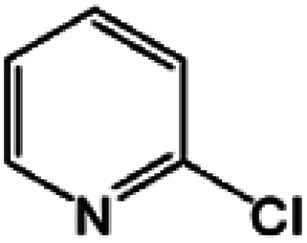	31	93
5	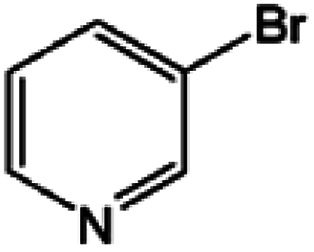	43	99
6	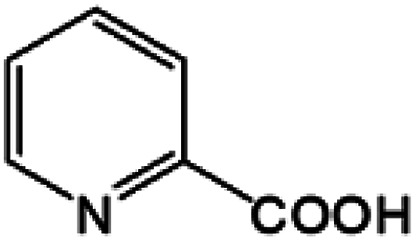	36	91
7	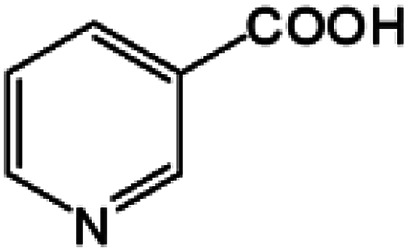	39	97
8	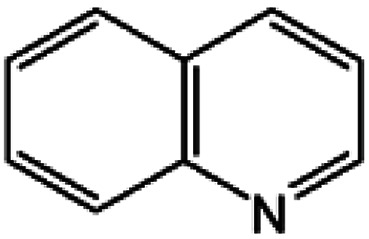	47	99

a Reaction conditions: pyridine (10 mmol), H_2_O_2_ (20 mmol, 15%), Od-MA (2.0 mmol).

b Determined by GC-MS.

### 3.5 Catalyst recyclability

**Fig. 7:**
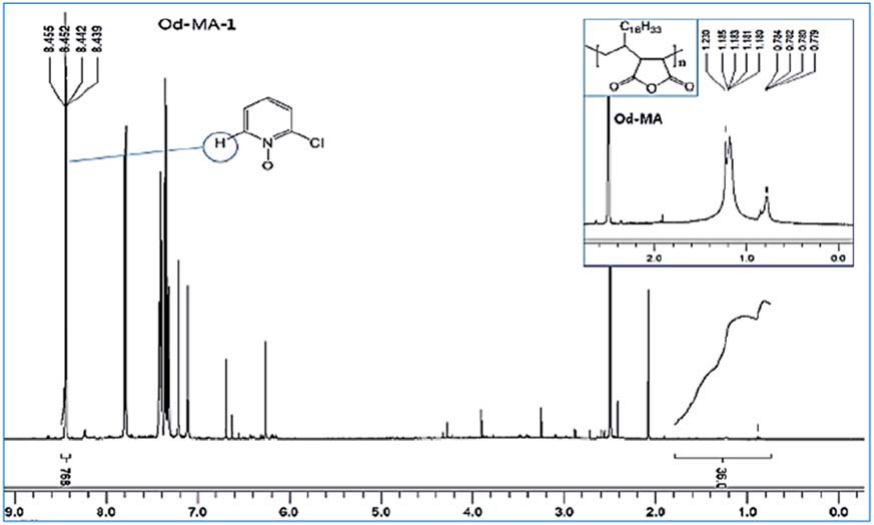
^1^H NMR spectrum of an aqueous extract of the reaction mixture after the first oxidation reaction using Od-MA.

Used catalysts were recovered *via* filtration of the aqueous reaction mixtures; recovered solid catalysts were reused without further treatment. Recovery yields of catalysts were determined by acquiring ^1^H NMR spectra of the aqueous filtrates after reaction completion. Filtrates were concentrated to obtain mixtures of CP, CPNO, and water-extracted Od-MA. In the ^1^H NMR spectrum of the concentrated mixture, the integration number (768) of the signal at 8.44 ppm corresponds to the 1H of CPNO (Fig. 7). The 36H of Od-MA appears at 0.80–1.70 ppm in the ^1^H NMR spectrum of Od-MA. Therefore, the molar ratio of Od-MA to CPNO was 1/768. In the first oxidation reaction, 0.2 eq. of Od-MA *versus* CP gave a CPNO yield of 93%. Therefore, the aqueous filtrate contained 2.4 × 10^−4^ [=0.93(0.2) (1/768)] equivalents of Od-MA *versus* CP, and the amount of recovered Od-MA was 0.2–2.4 × 10^−4^ eq. of CP (*i.e*., a 99.88% recovery yield, rounded off to 100%). Using the same procedure, recovery yields of Od-MA were all 100% after the 2^nd^, 3^rd^, and 4^th^ oxidation reactions (Table 3).

**Table 3:** Recovery yields of catalysts after repeated use

Catalyst	Recovery^c^^,^^d^ (%)			
	1^st^	2^nd^	3^rd^	4^th^
Od-MA^a^	100	100	100	100
Bu-MA^b^	93	92	89	—

a 0.2 eq. of Od-MA was used in the first reaction.

b 0.4 eq. of Bu-MA was used in the first reaction.

c Recovery yields were determined by ^1^H NMR.

d Reaction conditions: 15% of H_2_O_2_ (2.0 eq.), 90 °C, 7 h.

The recovery yields of Bu-MA were also determined based on the ^1^H NMR spectra of the filtrate after oxidation (ESI, S9–11†). Recovery yields of Bu-MA after the 1^st^, 2^nd^, and 3^rd^ reactions were 93%, 92%, and 89%, respectively. These results showed that the recovery yield of Od-MA was nearly quantitative after recycling. In contrast, the recovery yields of Bu-MA were lower than those of Od-MA and decreased slightly after each use. The excellent recovery yields of Od-MA are likely due to the hydrophobicity of the octadecyl side chain and the high molecular weight of Od-MA (average *M*_w_ = 40 000). Moreover, the reactivities of Od-MA and Bu-MA were retained, as indicated by the high % CPNO yields in Table 4, even after several cycles.

**Table 4:** Reactivities of recycled catalysts

Catalyst	CPNO yield^c^^,^^d^ (%)	
Cycle number	Od-MA^a^	Bu-MA^b^
1	93	90
2	94	91
3	94	91
4	93	—
5	94	—
6	94	—

a Amount of Od-MA: 0.2 eq. of CP.

b Amount of Bu-MA: 0.4 eq. of CP.

c Reaction conditions: CP (10 mmol), aqueous H_2_O_2_ (20 mmol, 15%), 90 °C, 7 h.

d Yields were determined by GC.

### 3.6 Application of Od-MA and H_2_O_2_ for the synthesis of zinc pyrithione

**Scheme 6:**
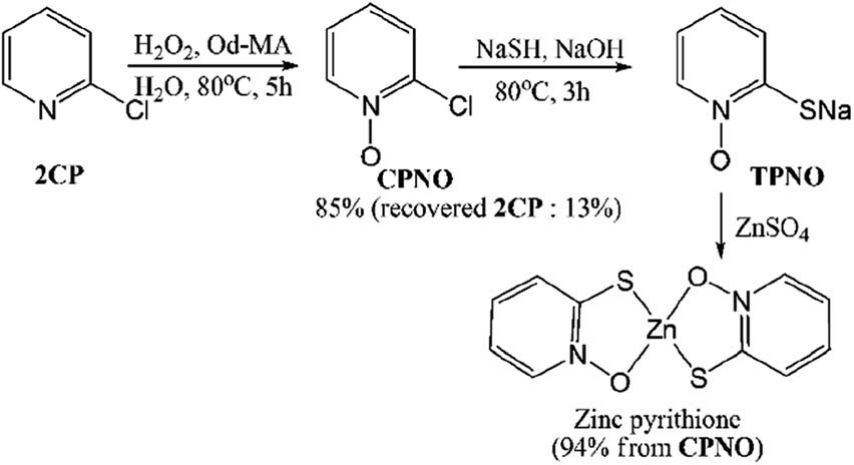
Synthesis of zinc pyrithione from 2CP.

Zinc pyrithione was synthesized from 2CP *via* the three steps shown in Scheme 6. After the oxidation of 2CP using Od-MA and H_2_O_2_, unreacted 2CP was recovered from the aqueous layer by extraction with toluene. Any remaining H_2_O_2_ in the aqueous layer was quenched with Na_2_S_2_O_3_. For the conversion of CPNO to sodium thiolate (TPNO), NaSH (1.3 eq.) and NaOH (1.3 eq.) were added to the aqueous CPNO solution and stirred for 3 h at 80 °C. The resulting thiolate (TPNO) was treated with ZnSO_4_ to form zinc pyrithione as a white solid.

It is noteworthy that the isolation of CPNO was not required and that the aqueous solution of CPNO was used directly in the subsequent reaction. In addition, the yield of the oxidation reaction was nearly quantitative, based on the amount of recovered unreacted 2CP. Used Od-MA was reused without further treatment. Thus, the Od-MA and H_2_O_2_ oxidation system described herein is suitable for the industrial production of zinc pyrithione.

## 4 Conclusions

Od-MA is capable of catalyzing the oxidation of pyridine derivatives to their corresponding *N*-oxides in the presence of H_2_O_2_ and used Od-MA can be easily recovered by filtration. Recovery yields of Od-MA exceeded 99.8% and the catalyst repeatedly retained its activity without further treatment between reactions. After filtration, aqueous solutions of CPNO could be used to synthesize zinc pyrithione. The described Od-MA and H_2_O_2_ catalytic system is eco-friendly, operationally simple, and cost-effective. It is suitable for the replacement of a wide range of oxidation reactions that depend on percarboxylic acid as an oxidant, particularly when the products are water-soluble.

## Conflicts of interest

There are no conflicts to declare.

## Supplementary Material

RA-010-D0RA00265H-s001
