# Supplementary material for: Recyclable anhydride catalyst for H2O2 oxidation: N-oxidation of pyridine derivatives
Source: RSC Adv. 2020 Mar 3;10(15):9165–71. doi: 10.1039/d0ra00265h (PMC9050160; doi:10.1039/d0ra00265h)
Supplement: RA-010-D0RA00265H-s001 [file RA-010-D0RA00265H-s001.pdf]

Electronic Supplementary Material (ESI) for RSC Advances.
This journal is © The Royal Society of Chemistry 2020

Supporting Information

Recyclable Anhydride Catalyst for H2O2 Oxidation: N-oxidation of Pyridine Derivatives

Ghellyn Gajeles, Se Mi Kim, Jong-Cheol Yoo, Kyung-Koo Lee, Sang Hee Lee*

Department of Chemistry, Kunsan National University, Gunsan 573-701, Republic of Korea

Corresponding author's e-mail: leesh@kunsan.ac.kr

^{1}H NMR and ^{13}C NMR of N-Oxide products:

2-carboxypyridine N-Oxide ($C_6H_5NO_3$)

δ ^{1}H NMR (500 MHz, DMSO): 7.88-7.95 (2H, m, Ar-H), 8.30-8.32 (1H, dd, J=2.5 Hz, 7.5 Hz, Ar-H), 8.73-8.74 (1H, m, Ar-H) ppm.

δ ^{13}C NMR (125 MHz, DMSO): 128.6, 130.1, 132.8, 135.9, 139.0, 160.9 ppm.

2-Chloropyridine N-Oxide (C_5H_4ClNO)

δ ^{1}H NMR (500 MHz, $CDCl_3$): 7.28-7.32 (2H, m, Ar-H), 7.55-7.58 (1H, m, Ar-H), 8.40-8.41 (1H, m, Ar-H) ppm.

δ ^{13}C NMR (125 MHz, $CDCl_3$ w/ DMSO): 123.8, 126.0, 126.9, 140.3, 141.5 ppm.

2-Methylpyridine N-Oxide

δ ^{1}H NMR (500 MHz, $CDCl_3$): 2.53 (3H, s, $-CH_3$), 7.20-7.32 (3H, m, Ar-H), 8.29-8.30 (1H, d, J = 5.5 Hz, Ar-H)

δ ^{13}C NMR (125 MHz, $CDCl_3$): 17.3, 123.2, 125.5, 126.1, 138.8, 148.5 ppm.

3-Bromopyridine N-Oxide

δ ^{1}H NMR (500 MHz, $CDCl_3$): 7.21-7.24 (1H, dd, J=6.6, 8.2 Hz, Ar-H), 7.45-7.47 (1H, dq, J=0.8, 8.3 Hz, Ar-H), 8.19-8.21 (1H, dq, J=0.8, 6.5 Hz, Ar-H), 8.39-8.40 (1H, t, J=1.5 Hz, Ar-H) ppm.

δ ^{13}C NMR (125 MHz, $CDCl_3$): 120.2, 125.9, 128.7, 137.7, 140.3 ppm.

4-Methylpyridine N-Oxide

δ ^{1}H NMR (500 MHz, $CDCl_3$): 2.37 (3H, s, -CH3), 7.12 (2H, s, Ar-H), 8.13 (2H, s, Ar-H) ppm.

δ ^{13}C NMR (125 MHz, $CDCl_3$): 20.1, 126.6, 138.0, 138.4 ppm.

Nicotinic N-Oxide

δ ^{1}H NMR (500 MHz, DMSO): 7.53-7.55 (1H, dd, J=6.8, 7.5 Hz, Ar-H), 7.76-7.78 (1H, d, J=8 Hz, Ar-H), 8.42-8.44 (1H, dd, J=0.7, 6.4 Hz, Ar-H), 8.48 (1H, s, Ar-H) ppm.

δ ^{13}C NMR (125 MHz, DMSO): 126.1, 127.2, 131.1, 139.4, 142.6, 164.7 ppm.

Pyridine N-Oxide

δ ^{1}H NMR (500 MHz, $CDCl_3$): 7.35-7.37 (3H, m, Ar-H), 8.25-8.27 (2H, m, Ar-H) ppm.

δ ^{13}C NMR (125 MHz, $CDCl_3$): 125.3, 125.5, 138.5 ppm.

Quinoline N-Oxide

δ ^{1}H NMR (500 MHz, $CDCl_3$): 7.29-7.32 (1H, dd, J=6.0, 8.5 Hz, Ar-H), 7.62-7.65 (1H, t, J=7.5 Hz, Ar-H), 7.74-7.77 (2H, m, Ar-H), 7.86-7.87 (1H, d, J=8.5 Hz, Ar-H), 8.54-8.56 (1H, d, J=6 Hz, Ar-H), 8.73-8.75 (1H, d, J= 9Hz, Ar-H) ppm.

δ ^{13}C NMR (125 MHz, $CDCl_3$): 119.4, 120.7, 126.2, 127.9, 128.5, 130.2, 130.3, 135.4, 141.1 ppm.

Zinc Pyrithione

δ ^{1}H NMR (500 MHz, DMSO): 6.99-7.02 (1H, td, J=2Hz, 7Hz, Ar-H), 7.23-7.26 (1H, m, Ar-H), 7.60-7.62 (1H x 2, dd, J=1.5, 8.5 Hz, Ar-H), 8.42-8.44 (1H x 2, dd, J=1.0, 6.5 Hz, Ar-H) ppm.

δ ^{13}C NMR (125 MHz, DMSO): 117.9, 128.4, 129.3, 137.3, 159.3 ppm.

Figures:

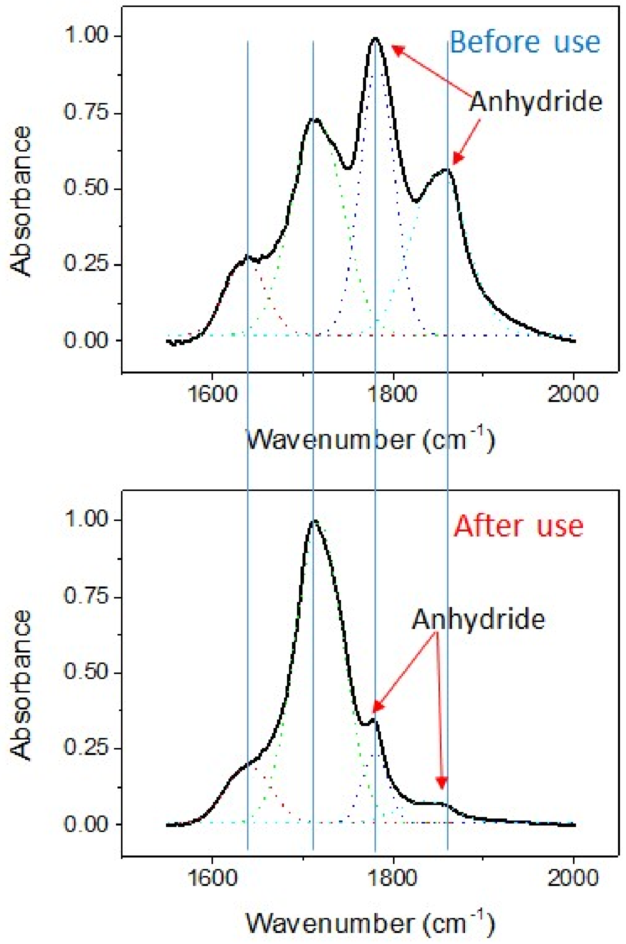

Wavenumber (Cm^{-1})	Area change (normalized based on 1637 Cm^{-1})	
	Before	After
1637	0.38	0.38
1716	1.00	1.81
1780	0.86	0.29<br>(34% of 0.86)
1859	0.84<br>(1.0)	0.23<br>(27% of 0.84)

Figure S1. IR spectrum of Od-MA before and after use in oxidation reaction.

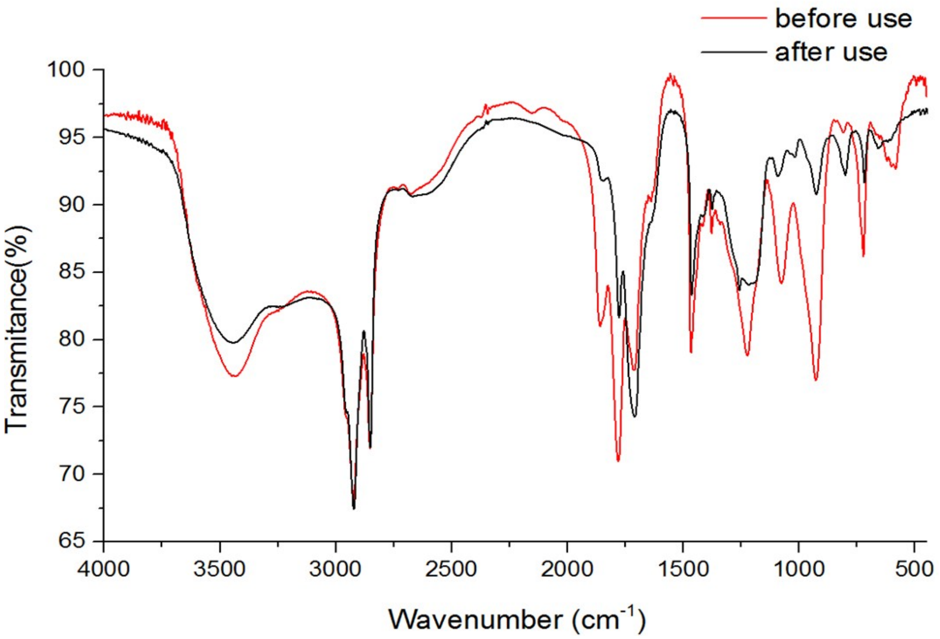

Figure S2. IR spectrum of Od-MA before and after use in oxidation reaction.

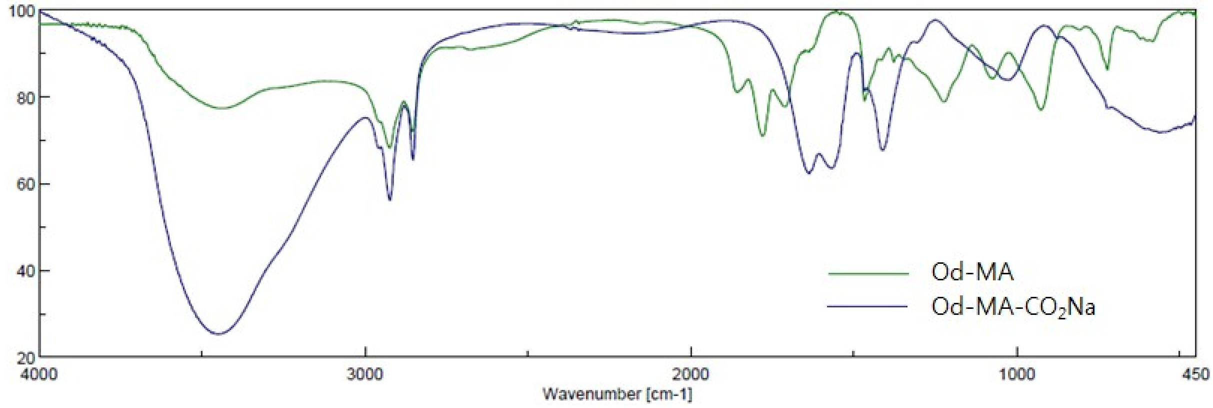

Figure S3. IR spectrum of Od-MA-CO_2Na.

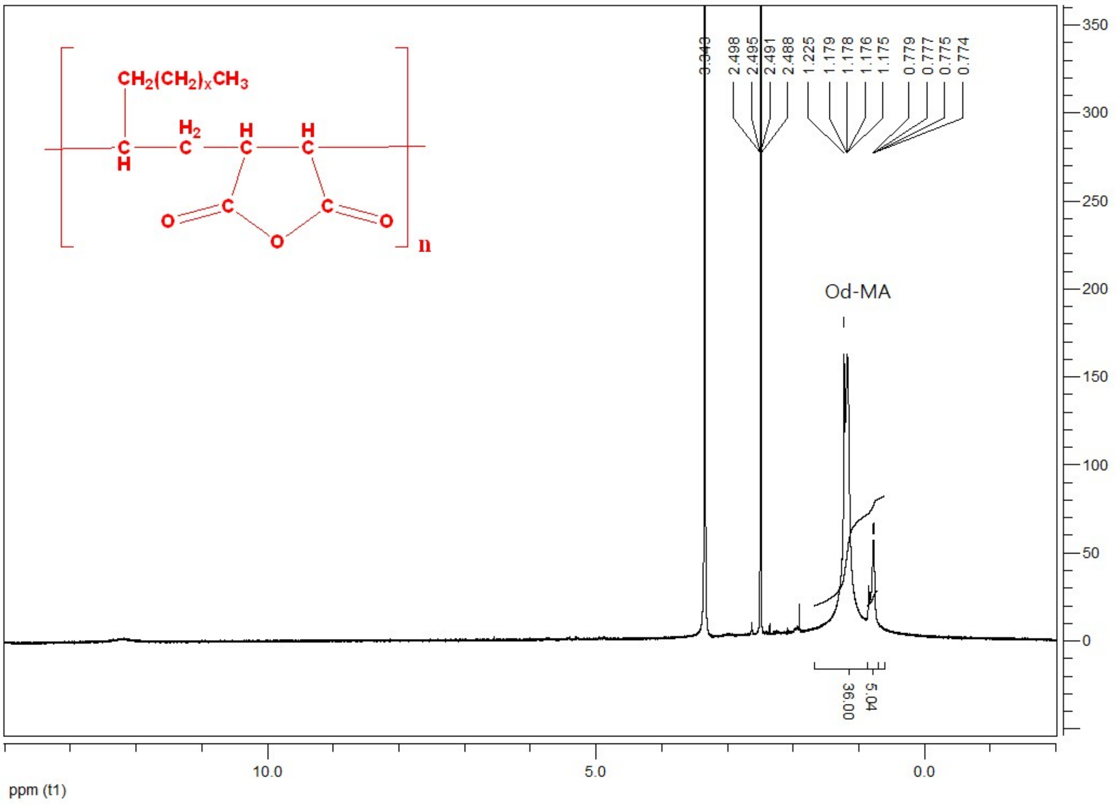

Figure S4. ^{1}H NMR spectrum of fresh Od-MA.

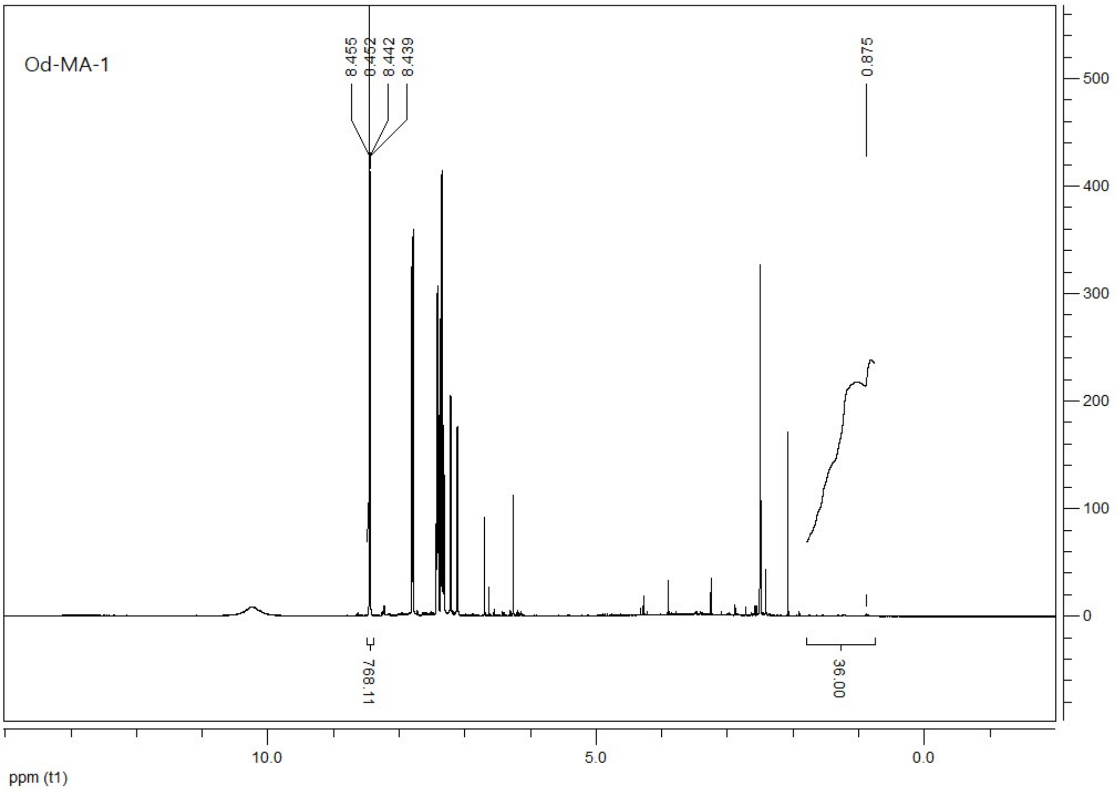

Figure S5. ^{1}H NMR spectrum of aqueous layer after 1st oxidation reaction using Od-MA.

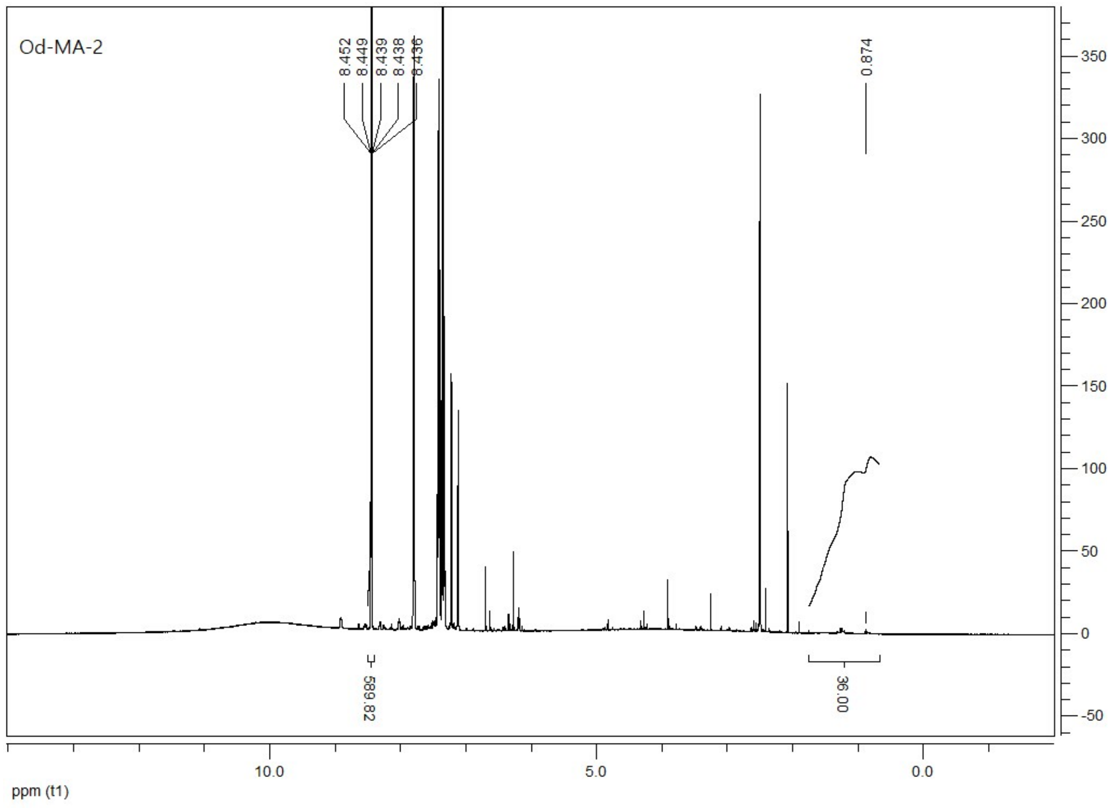

Figure S6. ^{1}H NMR spectrum of aqueous layer after 2nd oxidation reaction using Od-MA.

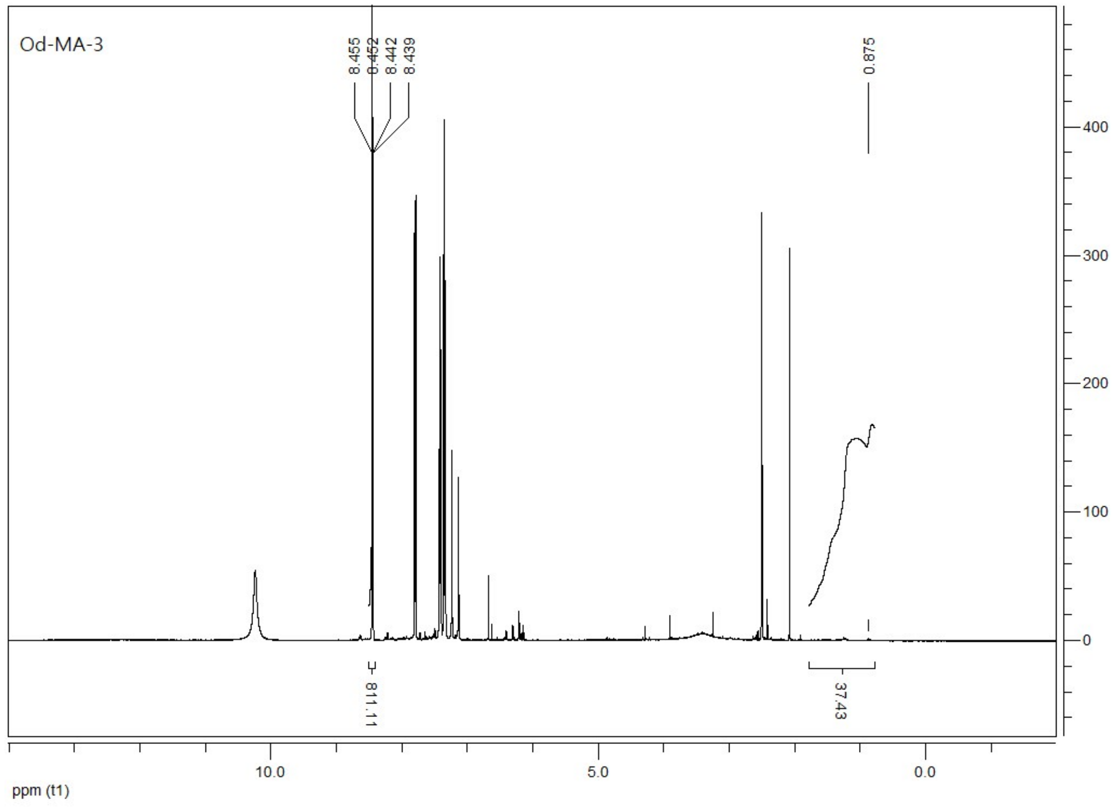

Figure S7. ^{1}H NMR spectrum of aqueous layer after 3rd oxidation reaction using Od-MA.

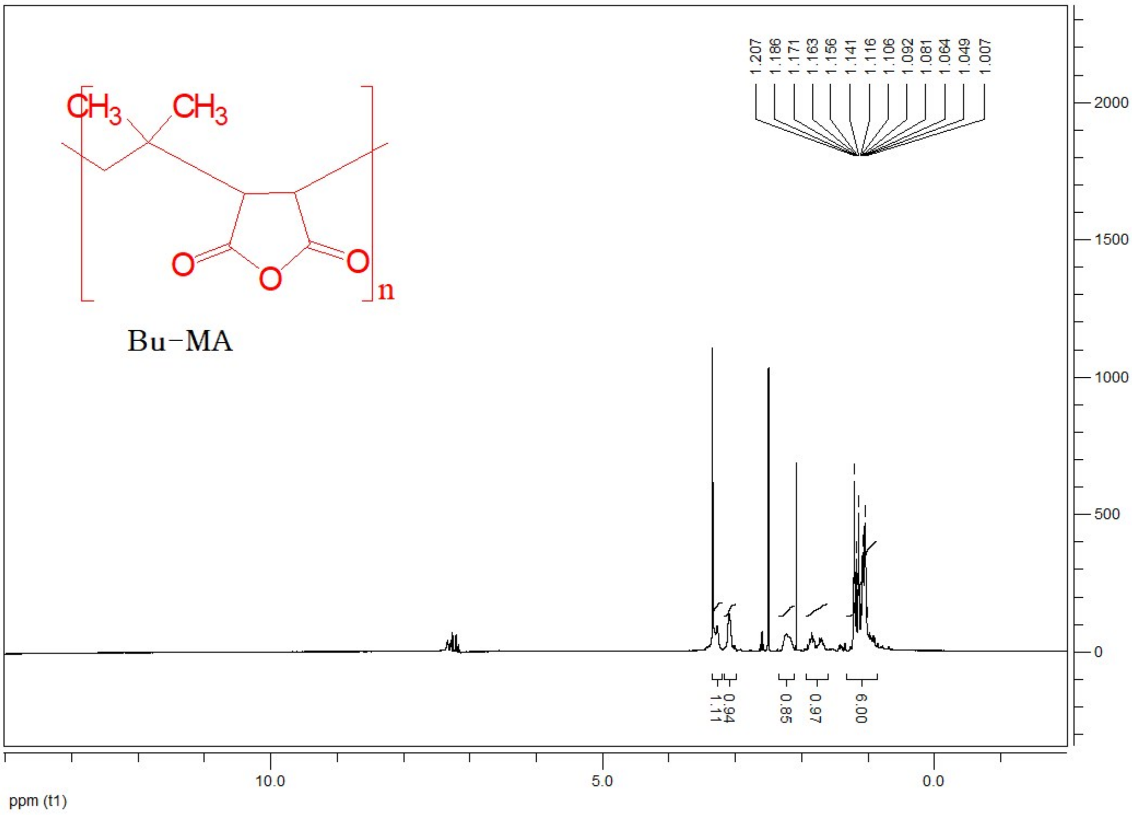

Figure S8. ^{1}H NMR (DMSO-d_6) of fresh Bu-MA.

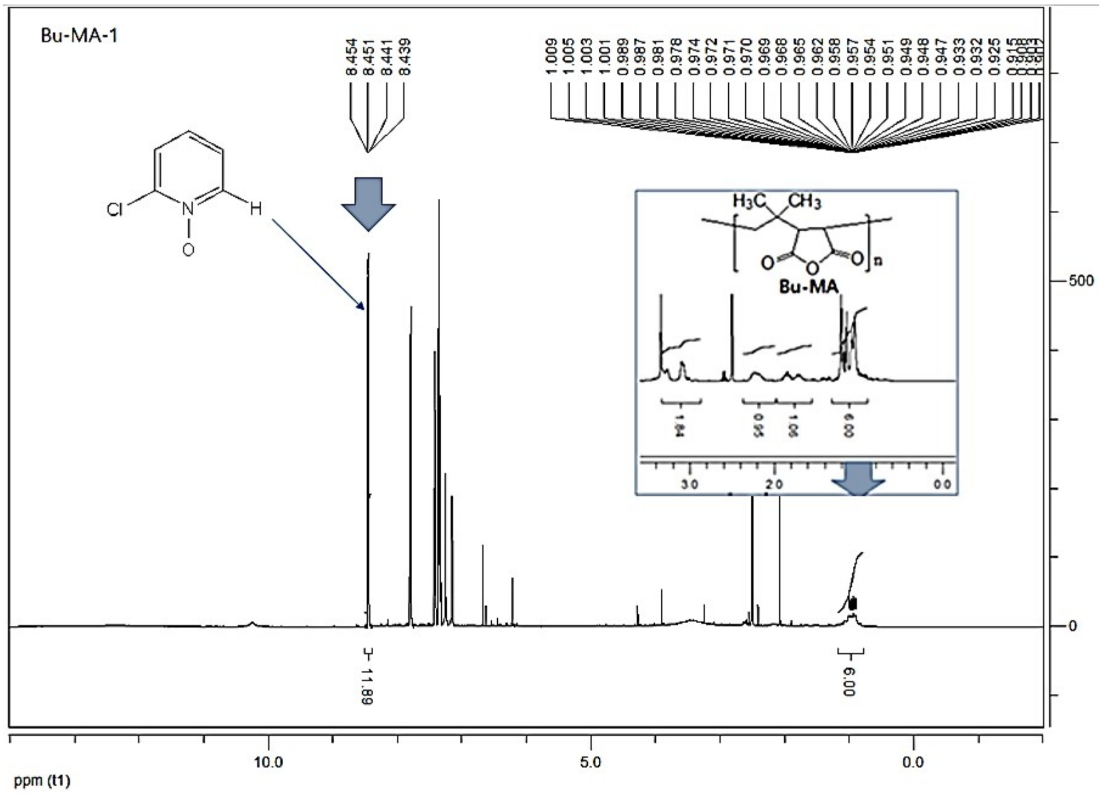

Figure S9. ^{1}H NMR spectrum of aqueous layer after 1st oxidation reaction using Bu-MA.

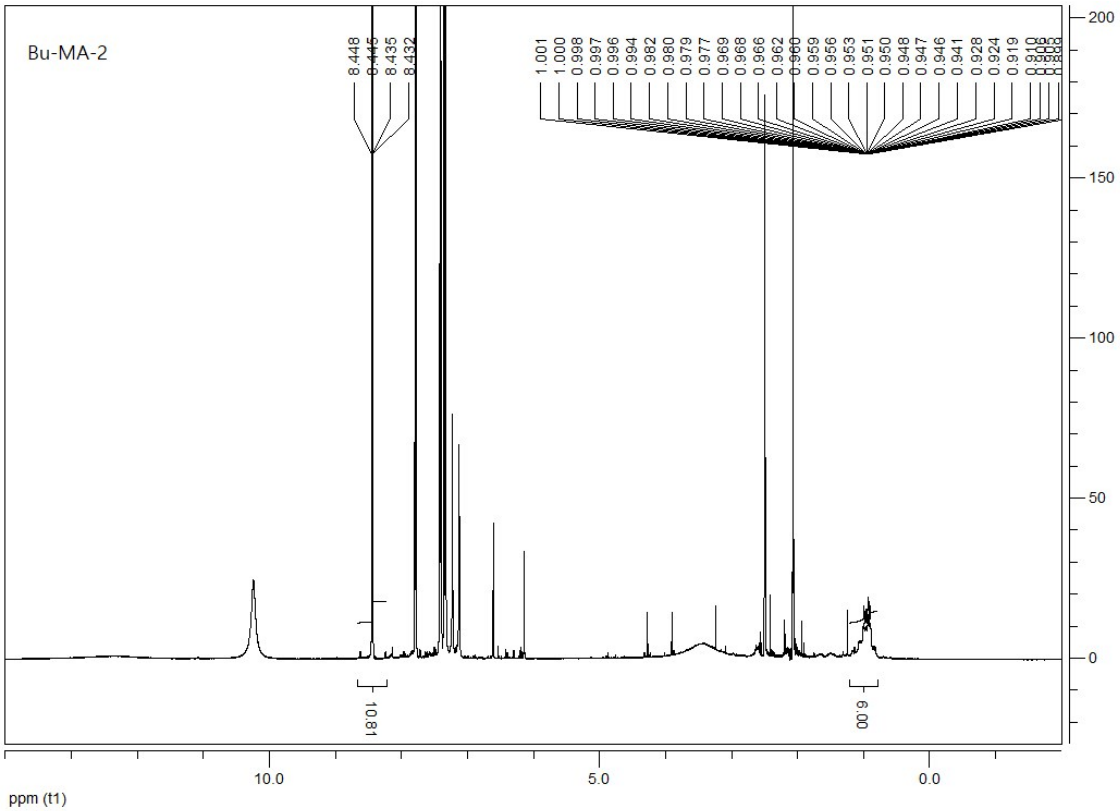

Figure S10. ^{1}H NMR spectrum of aqueous layer after 2nd oxidation reaction using Bu-MA.

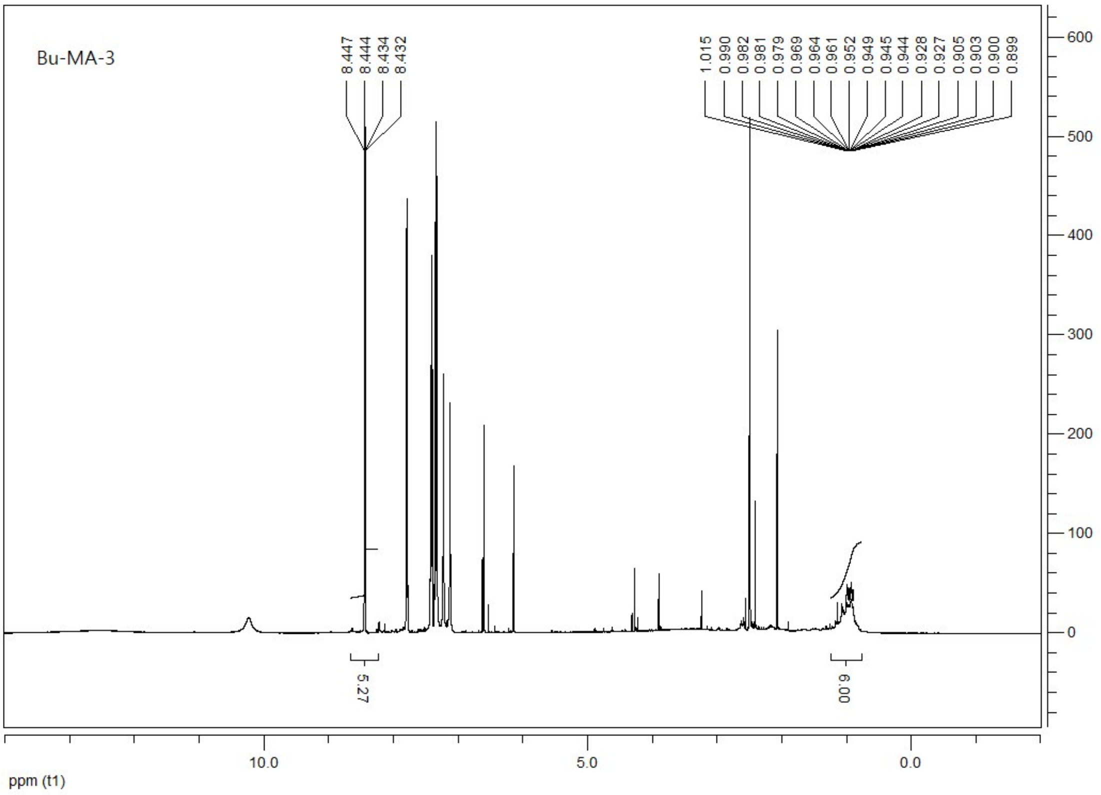

Figure S11. ^{1}H NMR spectrum of aqueous layer after 3rd oxidation reaction using Bu-MA.

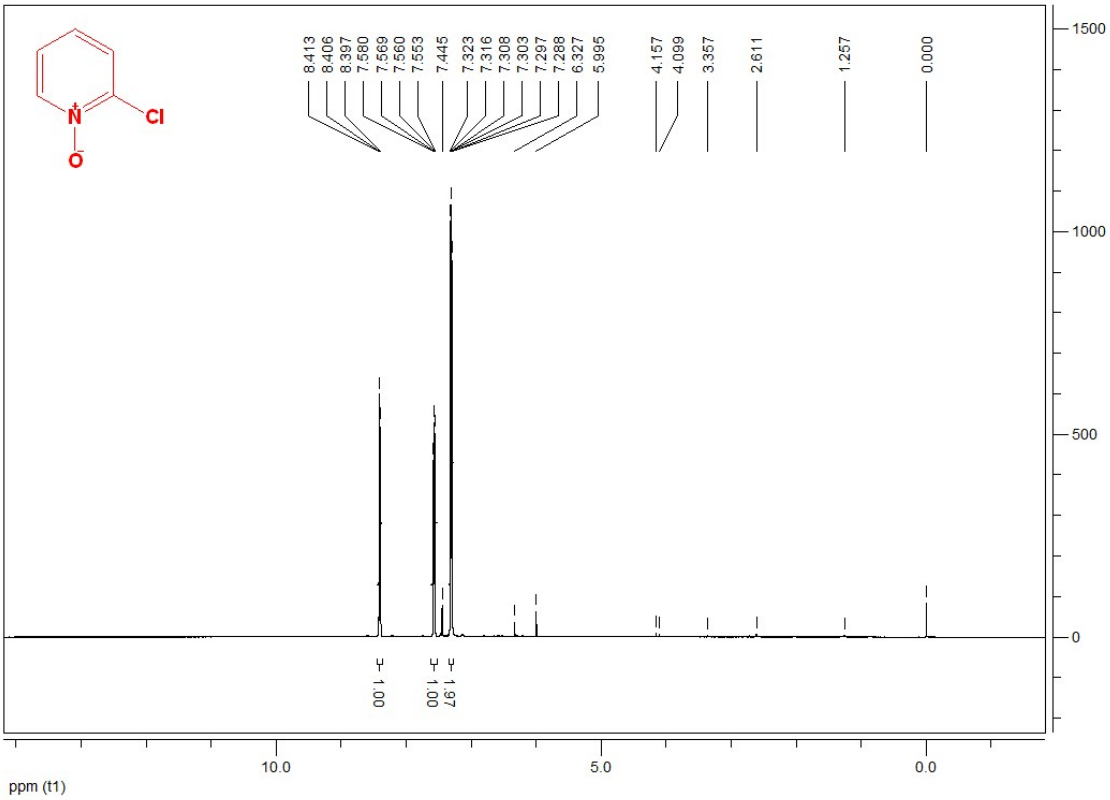

Figure S12. ^{1}H NMR spectrum of 2-Chloropyridine N-oxide.

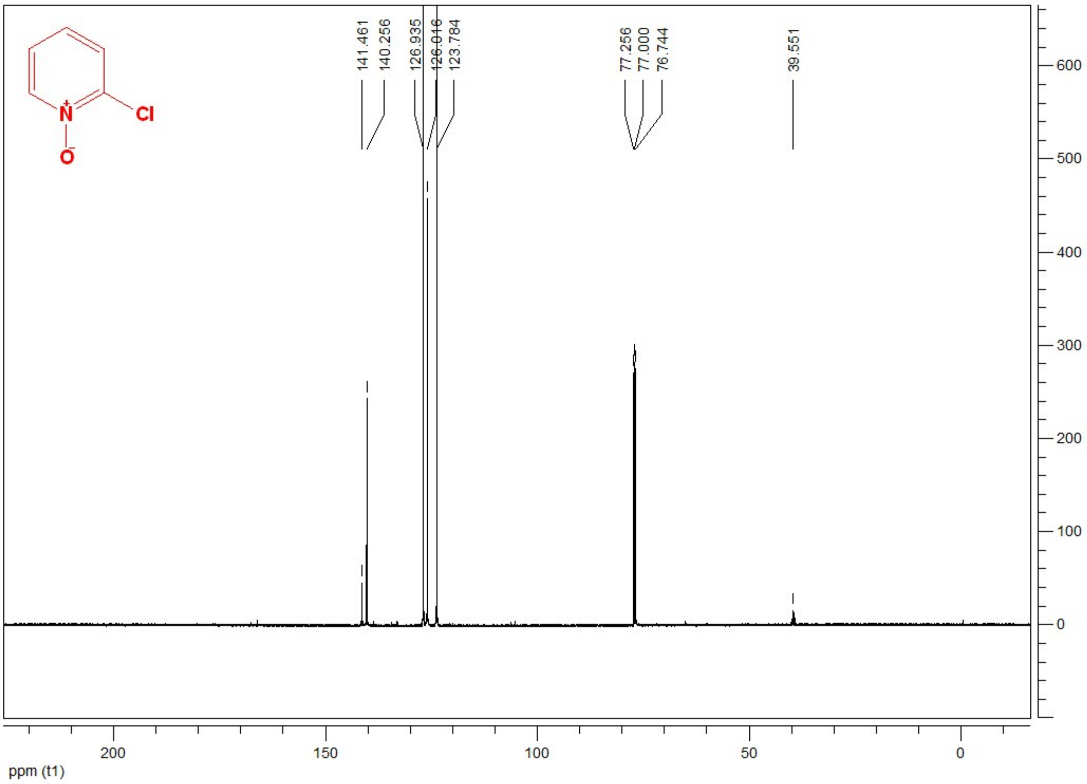

Figure S13. ^{13}C NMR of 2-Chloropyridine N-oxide.

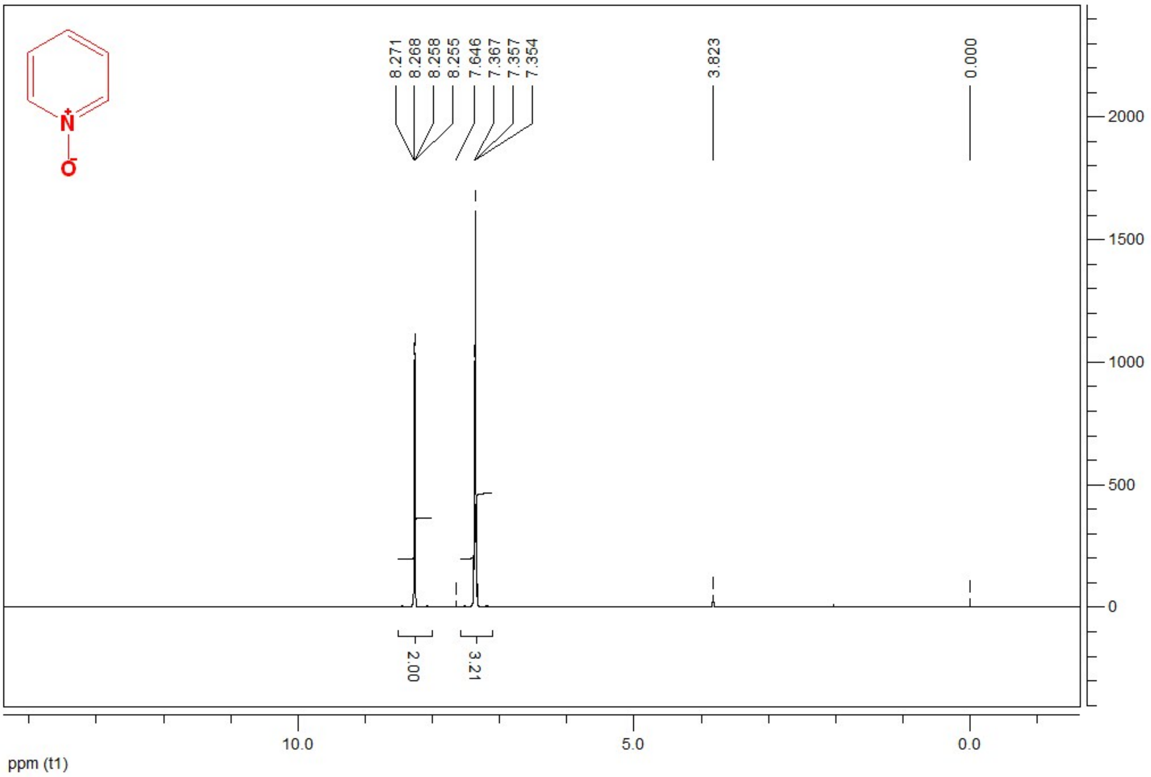

Figure S14. ^{1}H NMR spectrum of Pyridine N-Oxide.

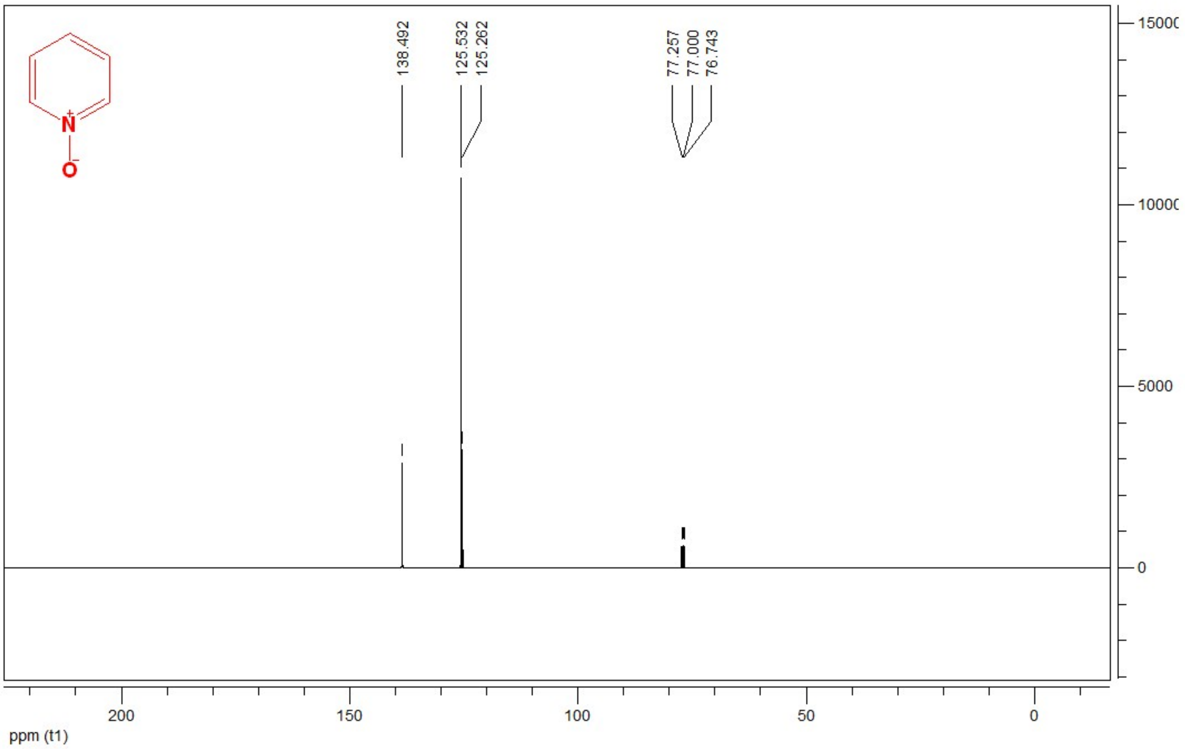

Figure S15. ^{13}C NMR spectrum of Pyridine N-Oxide.

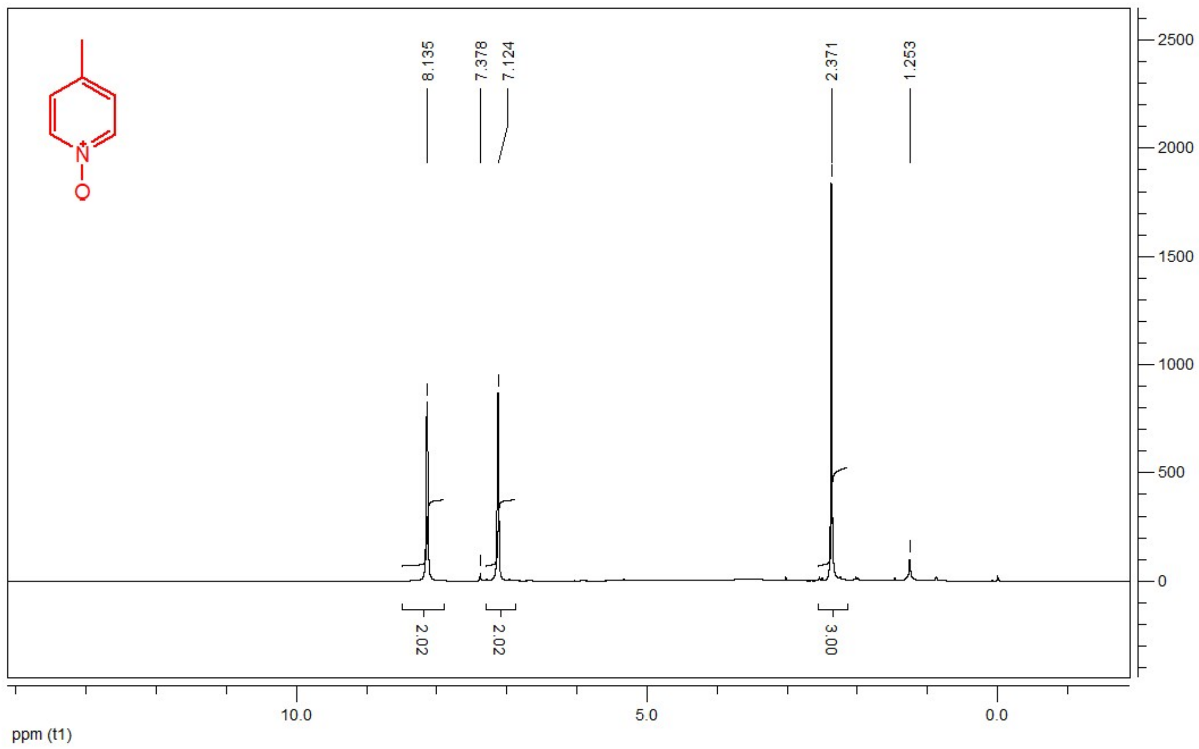

Figure S16. ^{1}H NMR spectrum of 4-Methylpyridine N-Oxide.

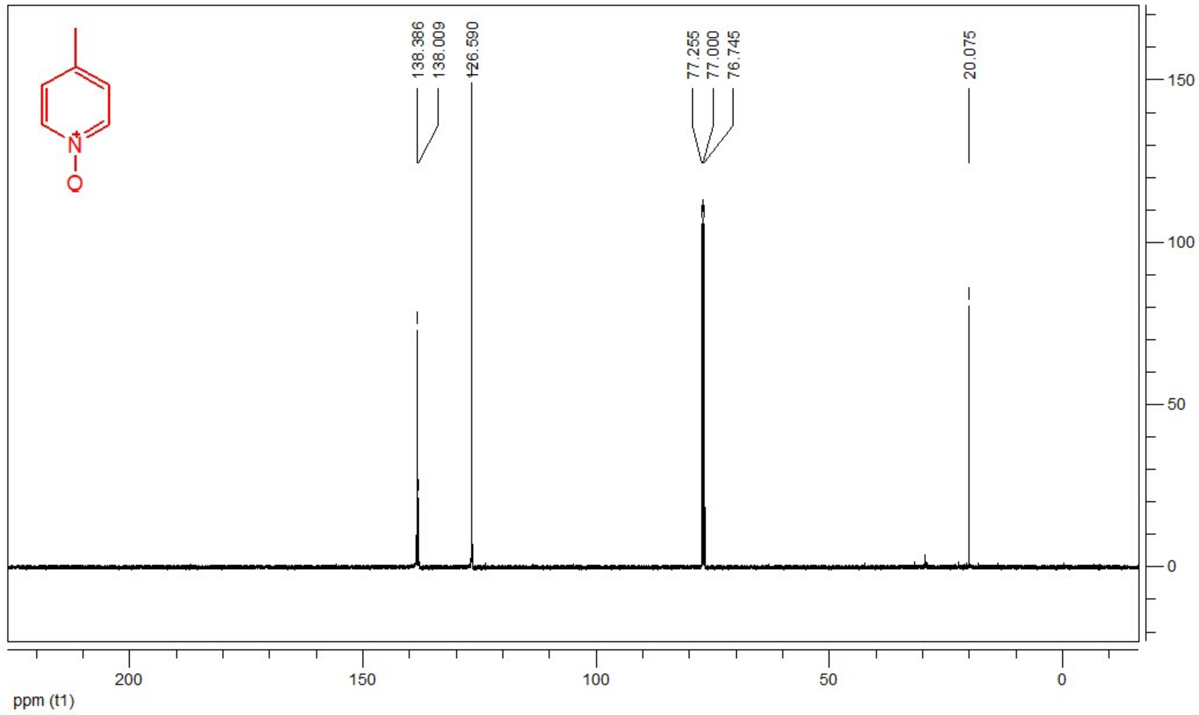

Figure S17. ^{13}C NMR spectrum of 4-Methylpyridine N-Oxide.

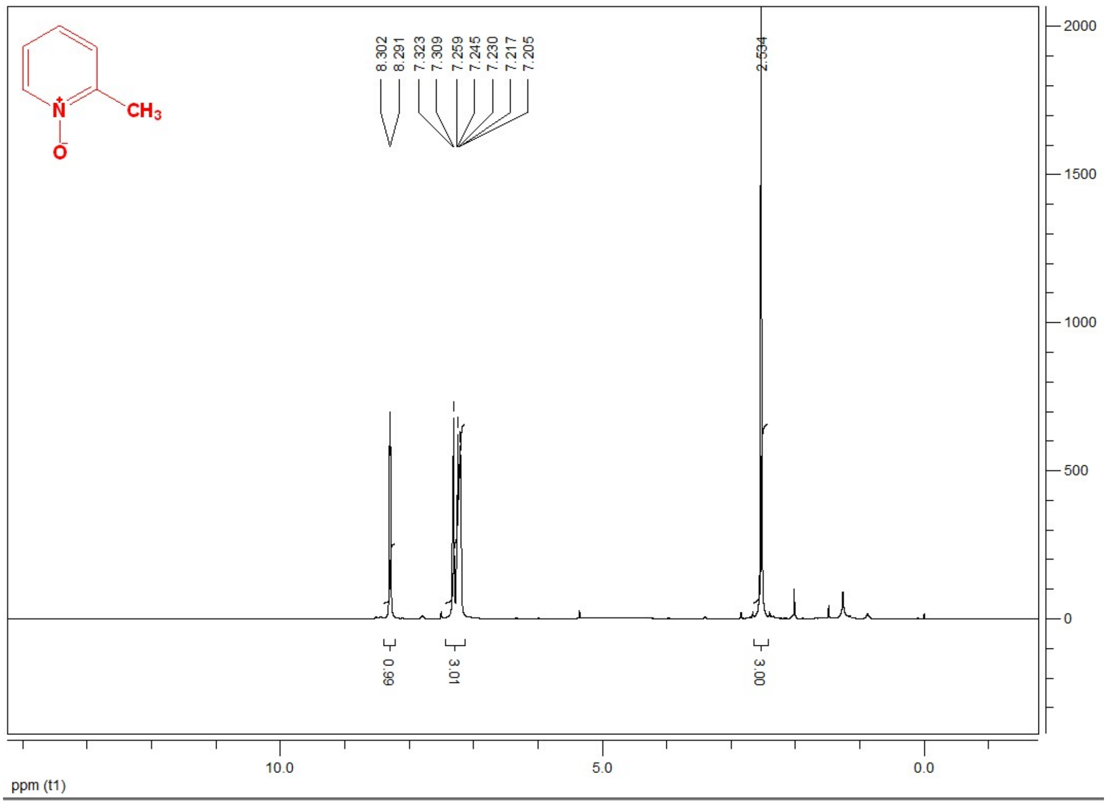

Figure S18. ^{1}H NMR spectrum of 2-Methylpyridine N-Oxide.

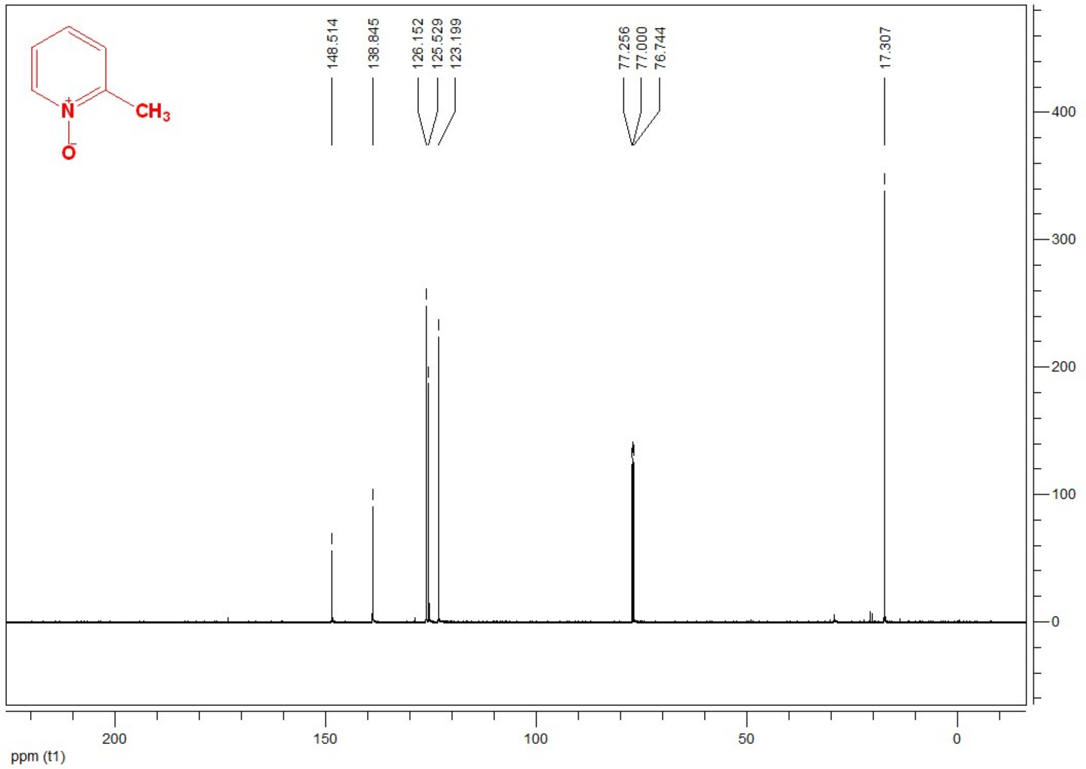

Figure S19. ^{13}C NMR spectrum of 2-Methylpyridine N-Oxide.

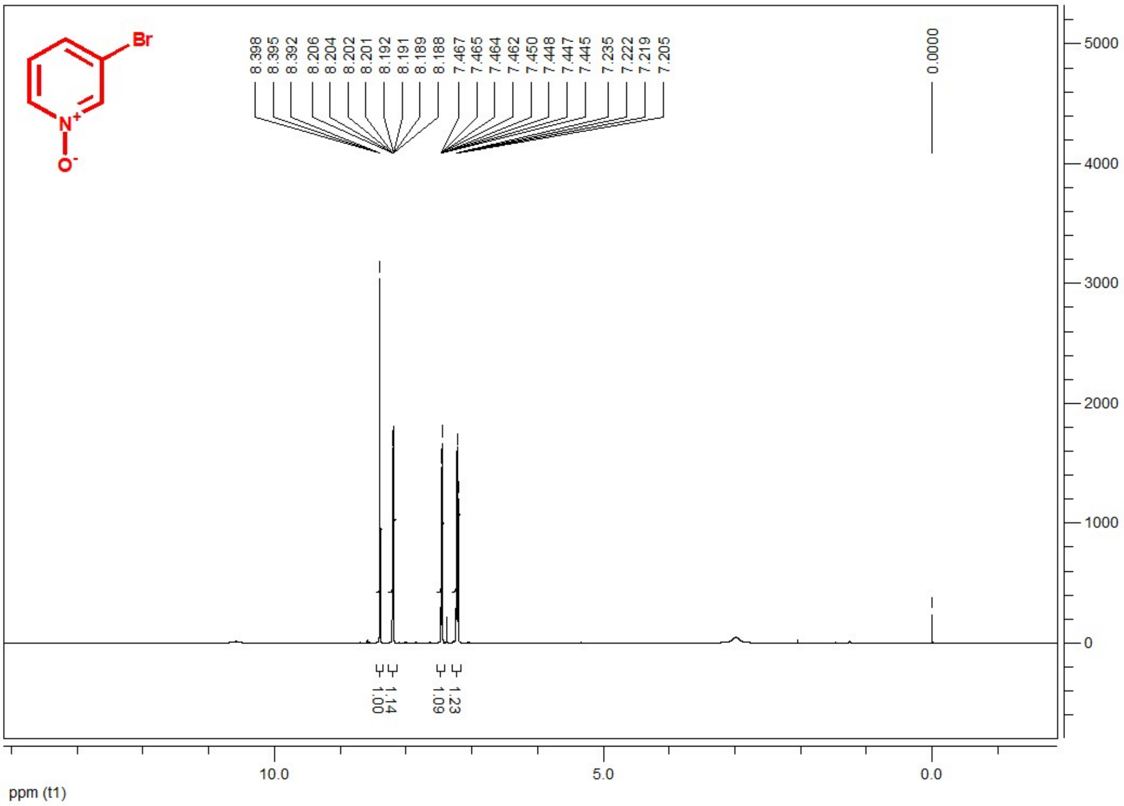

Figure S20. ^{1}H NMR spectrum of 3-Bromopyridine N-Oxide.

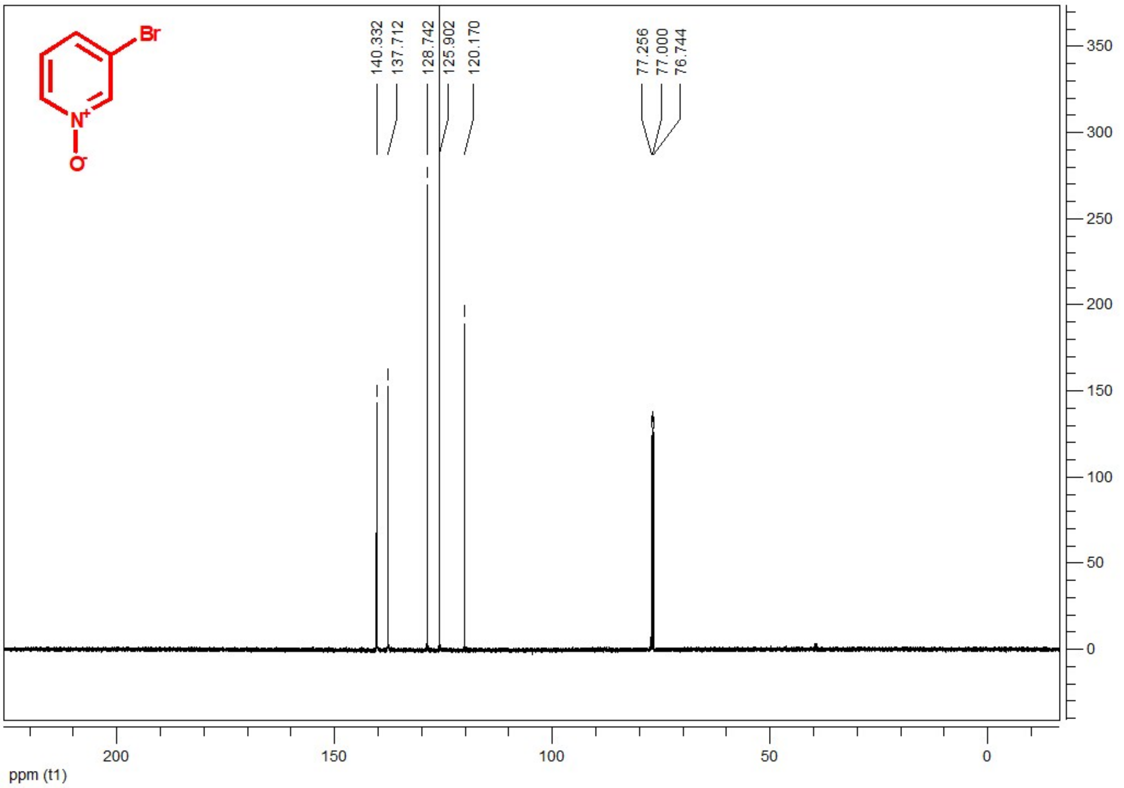

Figure S21. ^{13}C NMR spectrum of 3-Bromopyridine N-Oxide.

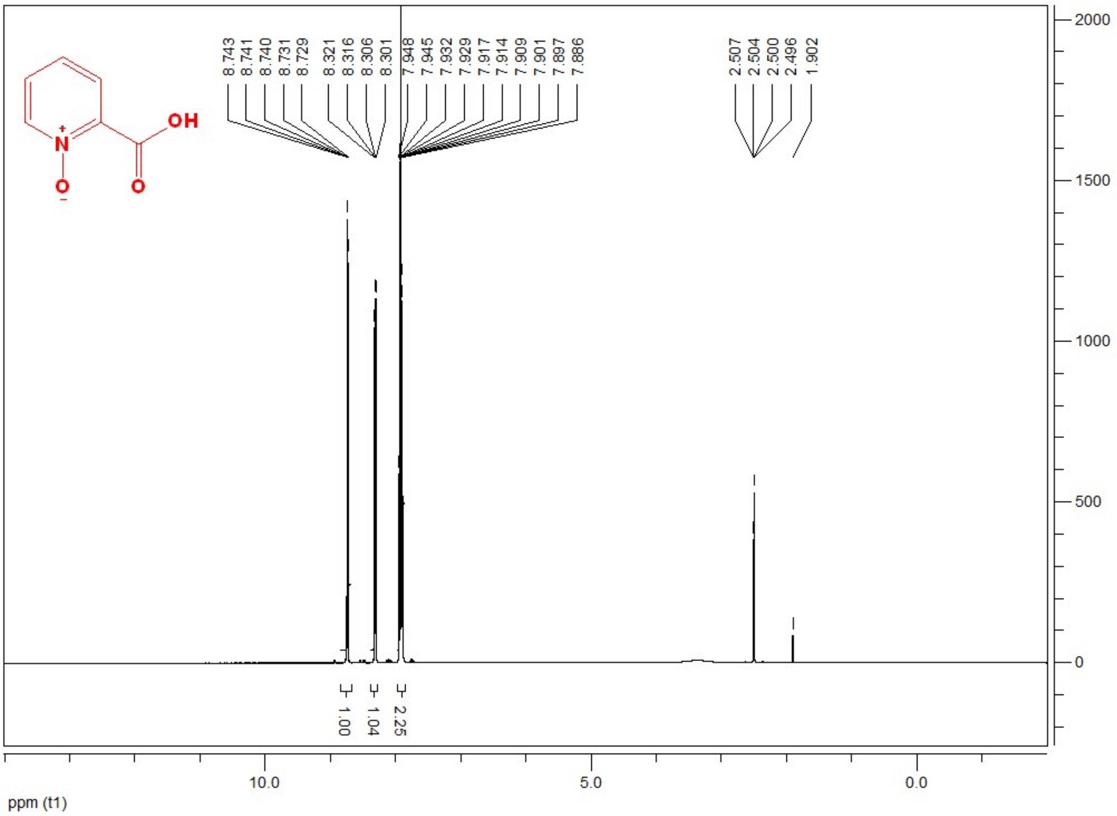

Figure S22. ^{1}H NMR spectrum of 2-Carboxypyridine N-Oxide.

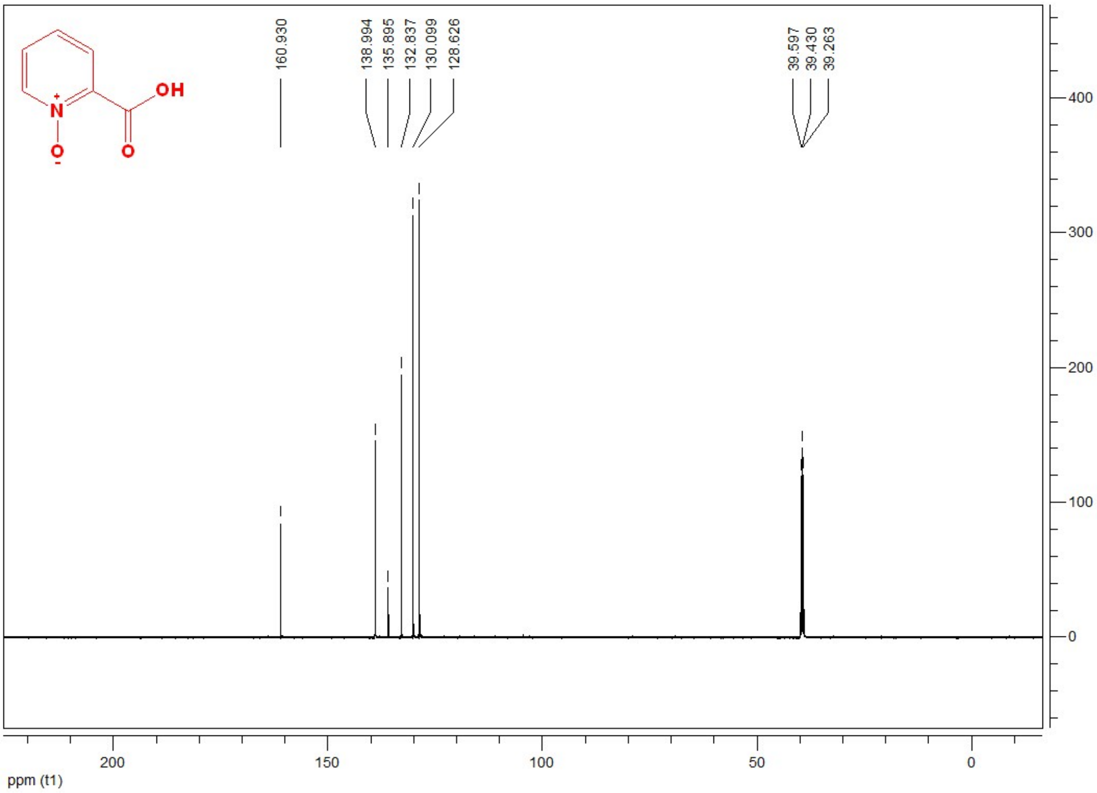

Figure S23. ^{13}C NMR spectrum of 2-Carboxypyridine N-Oxide.

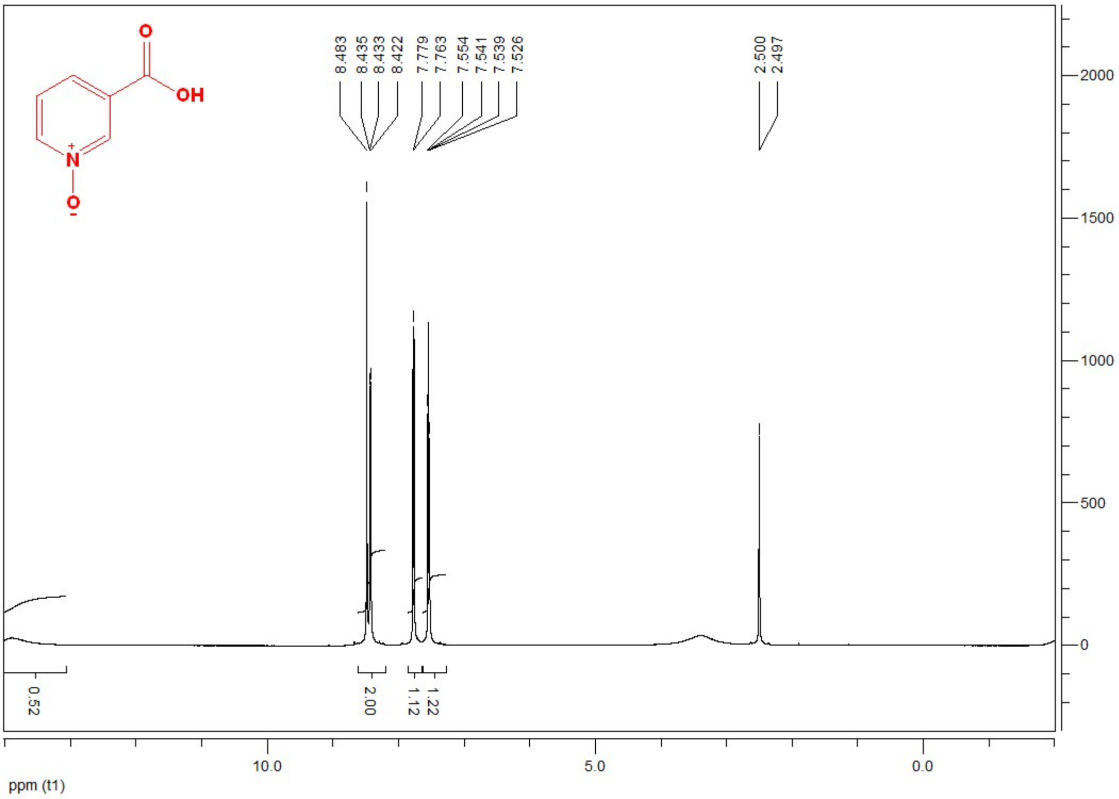

Figure S24. ^{1}H NMR spectrum of Nicotinic N-Oxide.

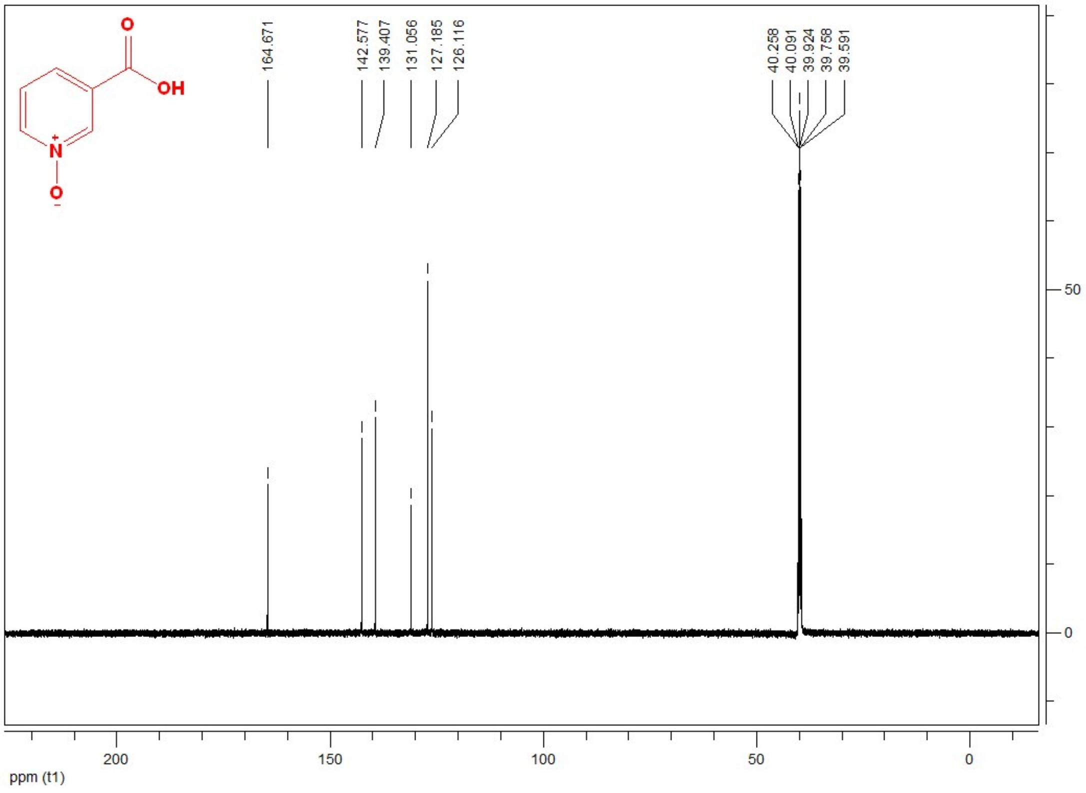

Figure S25. ^{13}C NMR spectrum of Nicotinic N-Oxide.

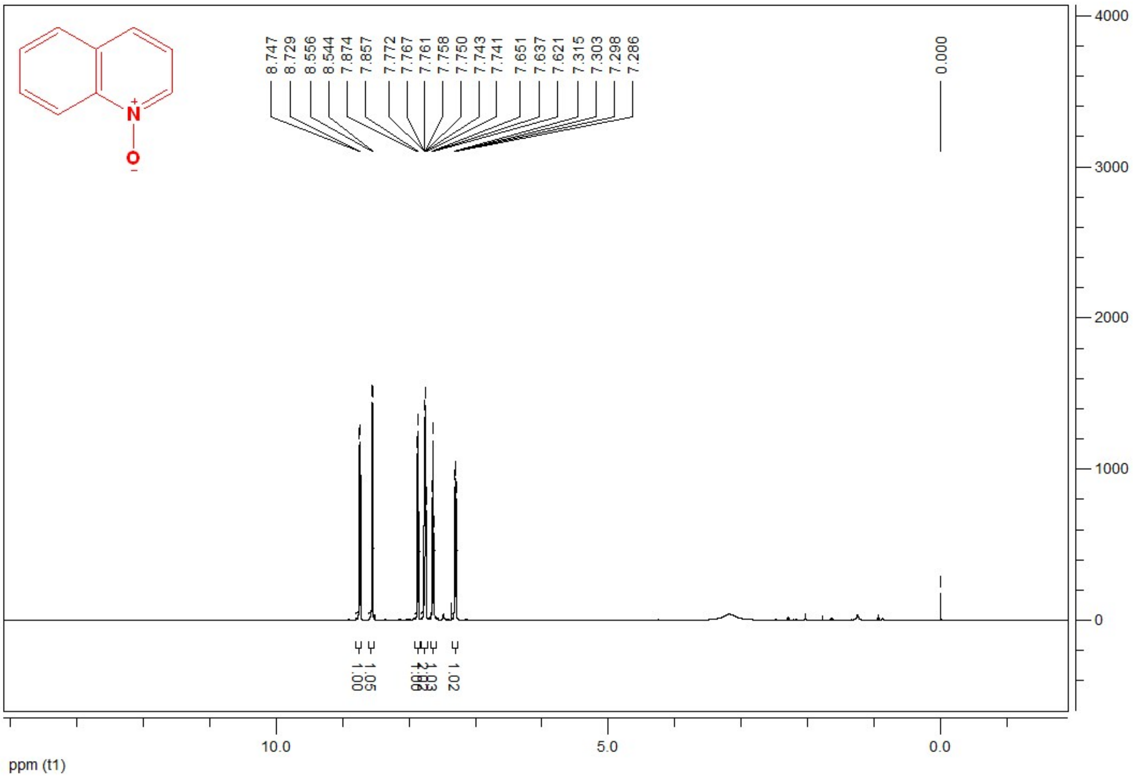

Figure S26. ^{1}H NMR spectrum of Quinoline N-Oxide.

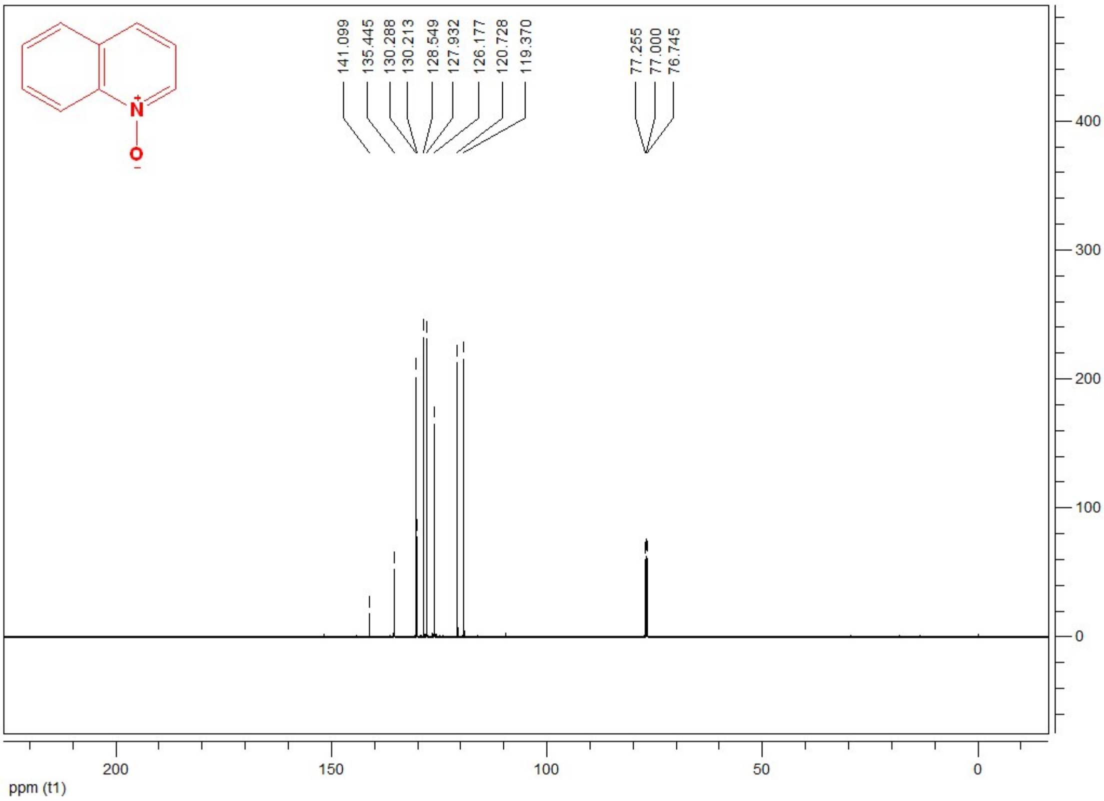

Figure S27. ^{13}C NMR spectrum of Quinoline N-Oxide.

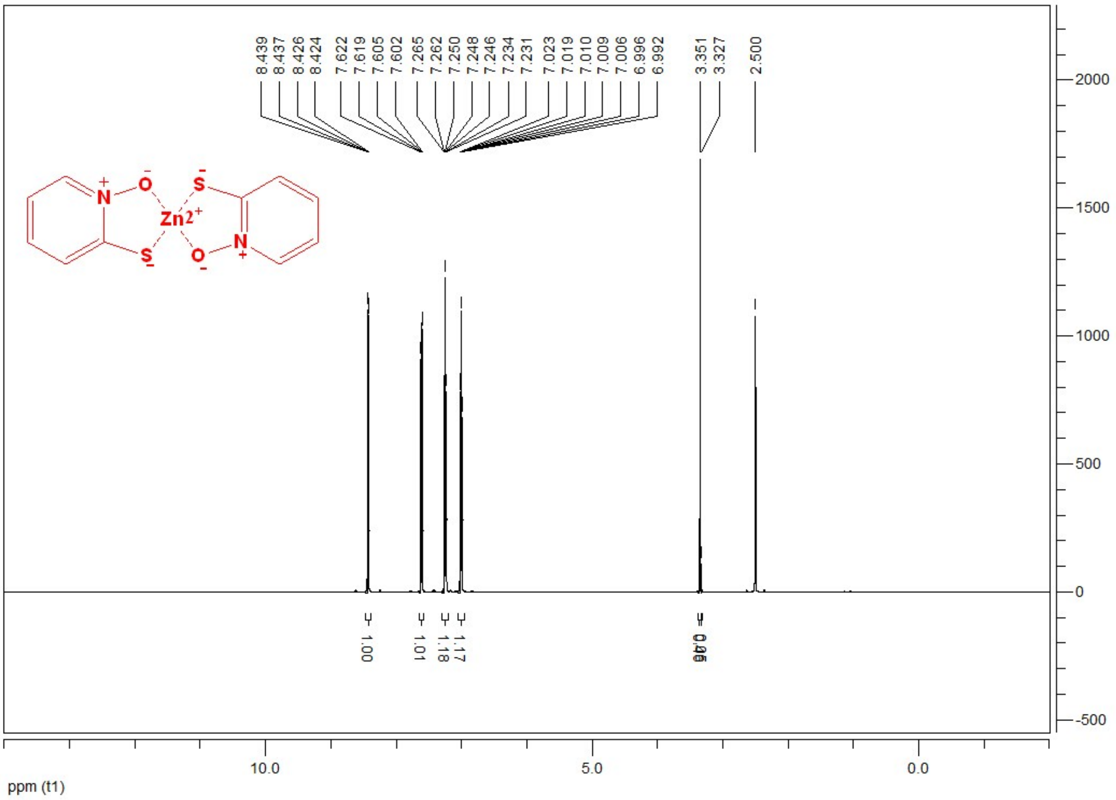

Figure S28. ^{1}H NMR spectrum of Zinc Pyrithione.

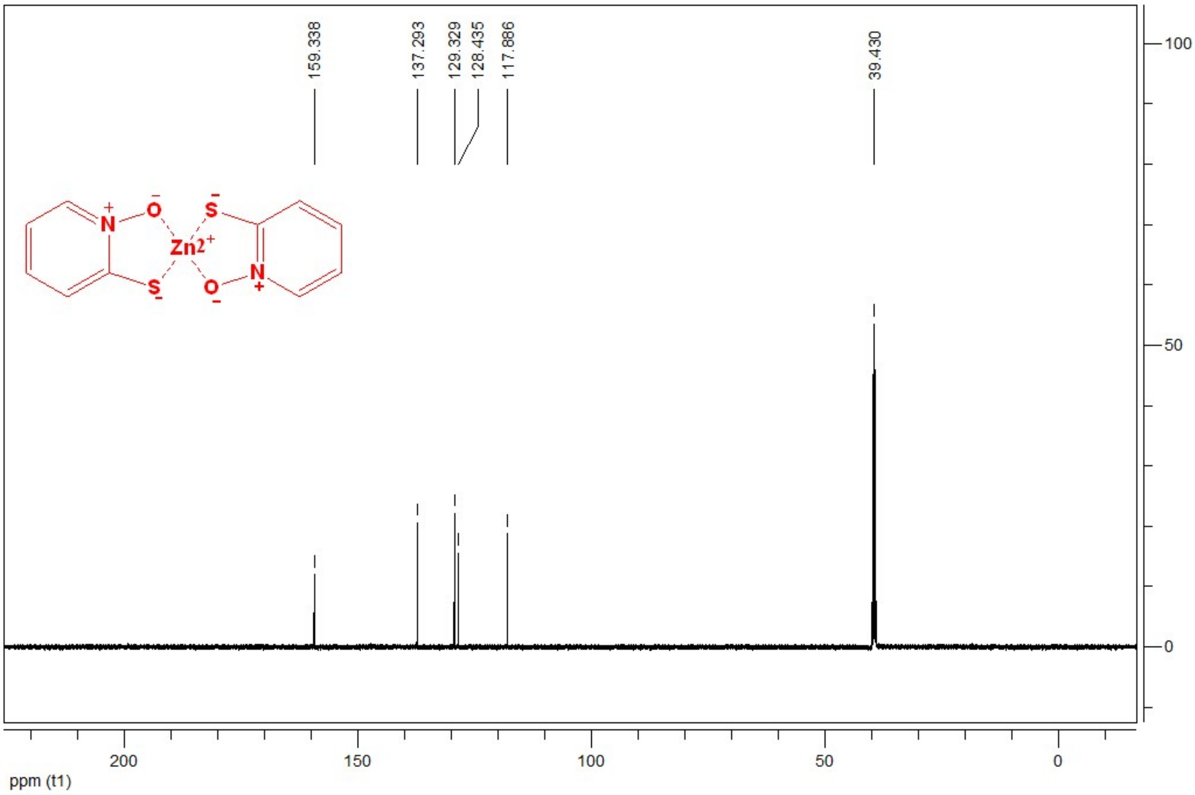

Figure S29. ^{13}C NMR spectrum of Zinc Pyrithione.
